# Analysis of structural diversity in wolf-like canids reveals post-domestication variants

**DOI:** 10.1186/1471-2164-15-465

**Published:** 2014-06-12

**Authors:** Oscar Ramirez, Iñigo Olalde, Jonas Berglund, Belen Lorente-Galdos, Jessica Hernandez-Rodriguez, Javier Quilez, Matthew T Webster, Robert K Wayne, Carles Lalueza-Fox, Carles Vilà, Tomas Marques-Bonet

**Affiliations:** Institut de Biologia Evolutiva (Universitat Pompeu Fabra - CSIC), Ciències Experimentals i de la Salut, Barcelona, 08003 Spain; Department of Medical Biochemistry and Microbiology, Science for Life Laboratory, Uppsala University, Uppsala, 75123 Sweden; Department of Ecology and Evolutionary Biology, UCLA, Los Angeles, 90095 CA USA; Estación Biológica de Doñana EBD-CSIC, Conservation and Evolutionary Genetics Group, Sevilla, 41092 Spain; Institució Catalana de Recerca i Estudis Avançats (ICREA), Barcelona, Spain; Centro Nacional de Análisi Genómico (CNAG), Barcelona, 08028 Spain

**Keywords:** Domestication, CNV, Candidate genes, Dog and wolf

## Abstract

**Background:**

Although a variety of genetic changes have been implicated in causing phenotypic differences among dogs, the role of copy number variants (CNVs) and their impact on phenotypic variation is still poorly understood. Further, very limited knowledge exists on structural variation in the gray wolf, the ancestor of the dog, or other closely related wild canids. Documenting CNVs variation in wild canids is essential to identify ancestral states and variation that may have appeared after domestication.

**Results:**

In this work, we genotyped 1,611 dog CNVs in 23 wolf-like canids (4 purebred dogs, one dingo, 15 gray wolves, one red wolf, one coyote and one golden jackal) to identify CNVs that may have arisen after domestication. We have found an increase in GC-rich regions close to the breakpoints and around 1 kb away from them suggesting that some common motifs might be associated with the formation of CNVs. Among the CNV regions that showed the largest differentiation between dogs and wild canids we found 12 genes, nine of which are related to two known functions associated with dog domestication; growth (*PDE4D*, *CRTC3* and *NEB*) and neurological function (*PDE4D, EML5*, *ZNF500*, *SLC6A11*, *ELAVL2*, *RGS7* and *CTSB*).

**Conclusions:**

Our results provide insight into the evolution of structural variation in canines, where recombination is not regulated by *PRDM9* due to the inactivation of this gene. We also identified genes within the most differentiated CNV regions between dogs and wolves, which could reflect selection during the domestication process.

**Electronic supplementary material:**

The online version of this article (doi:10.1186/1471-2164-15-465) contains supplementary material, which is available to authorized users.

## Background

The use of mtDNA, microsatellites, SNP arrays and whole genome sequencing has revealed some of the genetic changes underlying the generation of phenotypic diversity under domestication. Specifically a small set of genes associated with phenotypic traits related to morphology, coat texture, color and behavior have been identified that are common to breeds sharing a similar phenotype [1–5]. Other studies have also provided insight into the selective forces at play during the process of domestication [6–9], admixture with wild relatives [10, 11], or the population structure purebred and village dogs [12–14].

Structural variation refers to genomic alterations in the DNA content (insertions, deletions and inversions) greater than 50 bp in size [15]. Although fewer studies of structural variation have been performed in dogs compared to studies using SNPs or microsatellite loci, some examples of copy number variants (CNVs) that affect phenotype have been identified [2, 16, 17]. To date, four large-scale surveys of structural variation in dogs have been carried out using array comparative genomic hybridization (aCGH) [18–21], providing the first catalog of CNVs in the dog genome and candidate CNVs for breed-specific traits. However, very limited knowledge exists on the evolution and timing of CNV events.

A variety of genetic mechanisms affect CNV dispersion in humans [22], the most common mechanism being non-allelic homologous recombination (NAHR), which involves the misalignment and crossover between regions of extended homology during both meiosis and mitosis. In humans, the zinc-finger protein PRDM9 is implicated in the CNV formation by NAHR [23]. The inactivation of this gene in the canid lineage [24, 25] suggests that genomic features that promote the formation of CNV in canids might differ from the majority of mammals. Recently, Axelsson et al. [25] suggested that GC peaks represent novel sites of elevated recombination and genome instability in dogs, and Berglund et al. [21] proposed that these GC peaks were associated with the generation of many CNVs by NAHR events. However, the resolution of breakpoint in Berglund et al. was limited by the low density aCGH they used which precluded a fine-scale characterization of the regions. High-resolution approaches should provide new insight on the molecular mechanisms for CNV formation and dispersion in the genome. In addition, the analysis of outgroup species is needed in order to understand the origin and evolution of CNVs and their possible role in the origin of phenotypic diversity in domestic dogs. Specifically, the study of these loci in wolf-like canids, including the gray wolf (Canis lupus), the species from which domestic dogs derived, is needed to refine the assessment of ancestral states and variants that have appeared after domestication.

In this work, we designed a high density custom 720K probe aCGH chip to systematically genotype 1,611 CNVs derived mainly from modern dog breeds [20] in a new panel of 4 purebred dogs, one dingo (a feral Australian dog, presumably isolated from other dogs during thousands of years), 15 gray wolves from eleven genetically distinct populations worldwide (including Europe, Asia and America), one red wolf (*C. rufus*), one coyote (*C. latrans*) and one golden jackal (*C. aureus*). This expanded dataset of wolf-like canids, combined with a probe density higher than in previous studies, allowed us to perform the first high resolution characterization of CNVs in wolf-like canids and identify CNV break points over at a longer time-scale.

## Results and discussion

### Distribution and genomic effects of CNVs

To investigate CNVs in wolf-like canids we genotyped 23 canids (4 purebred dogs, one dingo, 15 gray wolves, one red wolf, one coyote and one golden jackal) for 1,611 CNVs previously typed in 61 dogs by Nicholas et al. [20] who compiled all the CNVs previously reported, mainly in modern dog breeds [18, 19] (Additional file 1: Table S1). We assessed the performance of our CNV genotyping using a two-stage procedure. In a first discovery stage, we identified CNVs using a conservative approach based on the combination on two methods: a Reversible Jump hidden Markov Model [26] and the procedure described in [21]. In the second stage, we genotyped our samples for each of these discovered CNV regions (see Methods).

We used three approaches to estimate false discovery rate and assess data quality. First, we performed two self-self hybridizations with a Boxer (the reference genome in our study) and a wolf from Iran. This analysis called only 12 and 11 CNVs, respectively, suggesting a low false discovery rate similar to that obtained by [20]. Second, we included 42 putative single copy control regions used by Nicholas et al. [20] on the aCGH chip. Across 966 control regions analyzed (42 regions × 23 samples), our algorithm only called 17 CNVs, suggesting a lower false discovery rate (1.75%) than obtained by [20]. Third, quantitative PCR (qPCR) was perfomed using Taqman assays on 10 canids (included the Boxer used in the aCGH experiments as a reference) to further validate 3 CNV regions (see Methods). In all the cases the qPCR validate the CNV regions. Assuming the qPCR results represent the correct copy number of individuals, we estimate a false positive rate of 0 and a false negative rate of 17.66% in the calling in the aCGH data, confirming the conservativeness of threshold for calling CNVs in the aCGH data.

**Figure 1:**
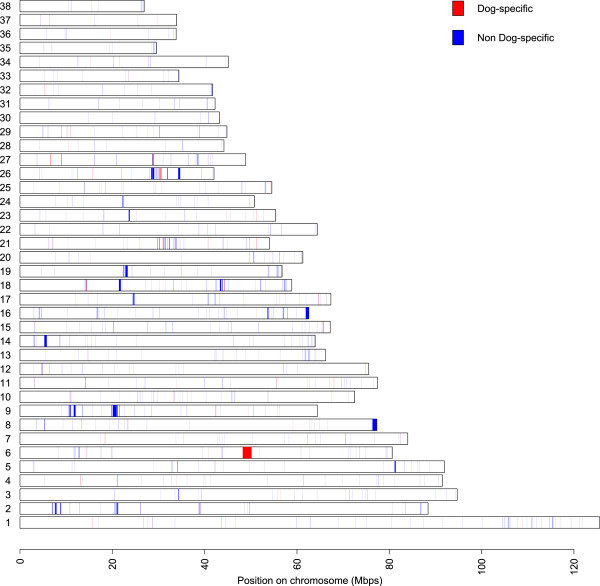
**Chromosomal distribution of 860 CNVs in the canine genome.** The chromosomes are represented by bars; colors indicate the location of the 860 CNVs. Red marks indicate dog-specific CNVs.

We found a total of 860 CNVs distributed in 715 of the 1,611 regions analyzed (Figure 1, Table 1, Additional file 2: Table S2). Many of the regions analyzed (55.6%) did not show any CNV in our dataset probably due to several reasons. First, not all the previously reported CNVs had the same level of support. In fact, only 31.28% of the original 1,611 regions previously analyzed were labeled as “high confidence CNVs” (as reported in [20]) and we found CNVs in our dataset in almost 75% of these regions. Second, the design of the array was based almost exclusively on modern dog breeds (26 dogs from 21 breeds and only one wolf) and a high proportion of the CNVs were identified in just one individual each (32% in [19] and 64.5% in [20]). Since we only genotyped 4 purebred dogs, many of these CNVs may not have been detectable.

**Table 1:** **Summary of CNVs genotyped per sample**

Sample	Total CNVs			Unique CNVs*		
	Total	Gains	Loses	Total	Gains	Loses
Boxer	153	88	65	19	8	11
Dachshund	218	92	126	7	2	5
Beagle	209	89	120	16	7	9
Basenji	267	90	177	32	8	24
Dingo	186	56	130	8	0	8
IsraelWolf	252	128	124	3	0	3
IsraelWolf2	194	75	119	2	0	2
ItalianWolf	224	100	124	0	0	0
ItalianWolf2	185	89	96	2	0	2
PortugueseWolf	202	77	125	5	2	3
IberianWolf	211	95	116	6	4	2
YellowstoneWolf	270	79	191	34	2	32
GreatlakesWolf	268	148	120	12	10	2
IranianWolf	209	80	129	0	0	0
MexicanWolf	211	93	118	2	0	2
ChineseWolf	181	73	108	0	0	0
IndianWolf	226	87	139	9	4	5
MongolianWolf	162	71	91	0	0	0
MongolianWolf2	265	136	129	14	9	5
SwedenWolf	224	94	130	10	7	3
RedWolf	223	123	100	8	6	2
Coyote	200	94	106	4	1	3
Golden Jackal	215	103	112	22	4	18

*Unique are defined as CNV that are present only in one sample.

Of the 860 CNVs regions that we identified, 412 (47.9%) were shared between dogs and wild canids. Dog-specific CNVs were 12.3% (106 CNVs) of the total but the design of the array and the different number of samples analyzed (5 *vs* 18) suggests this was an underestimation (Figure 1). These 106 derived CNVs may have originated after domestication but most of them (78.3%) were present in only one dog, so likely arose later in the evolutionary history of dogs. Selection could have fixed some of these variants in some breeds or alternatively, given the small effective population size of breeds, strong genetic drift and founder effect might have overcome the possible negative effects of CNVs. Consequently, we analyzed whether these 106 CNVs were enriched for genes, compared to the 754 non-dog-specific regions (860–106) or to the total 1,611 regions (see Methods). Although not all intergenic variants may be neutral (for example, by influencing the expression levels of nearby genes [27]), our randomization test suggested that those 106 CNVs might not be under strong selection since we did not find any enrichment in the number of genes in dog specific regions compared to non-dog-specific regions (*P*-value = 0.744) or the total 1,611 regions (*P* = 0.844) (Additional file 1: Figure S1). Similarly, no gene ontology category was overrepresented in dog-specific or in the whole set of 1,611 CNV regions.

In relation to overall CNV diversity, the sample with lowest CNVs identified was the Boxer, probably because the reference was also a Boxer. In the same way, we also found more CNVs in wolves than in dogs (Table 1). In order to quantify the differences between dogs and wolves, we calculated allele frequencies for each CNV in dogs and wolves using the EM algorithm [28]. From these allele frequencies, we estimated the expected heterozygosity (H_e_) for each polymorphic CNV and the average for dogs and gray wolves. Since the number of wolf samples analyzed was higher (15 gray wolves *vs* 5 purebred plus dingo), we estimated the random expectation averaging H_e_ for 1,000 groups of 5 randomly selected gray wolves and found that the structural variability in dogs and gray wolves are very similar (0.299 ± 0.009 for wolves vs 0.305 for dogs, *P* = 0.235). Domestication is associated with a very large reduction in the population size in dogs (16-fold compared to a much smaller 3-fold reduction in wolves; [29]). However, we do not see a similar reduction of CNV variation in dogs in our aCGH data, most likely because of the ascertainment bias in the design of the array, which is expected to result in higher levels of CNV variation in dogs.

In agreement with previous studies [18–21, 30, 31], we found more losses than gains both in dogs and wolves. This is partly attributable to technical biases, because in aCGH experiments copy gains are more difficult to genotype than losses [21]. Since in aCGH experiments losses and gains are relative to the reference genome it is not possible to separate duplications and deletions without an outgroup. We used data from wolf-like canids to determine the ancestral state and thus identify duplications and deletion in dogs. We considered a post-domestication CNV event any gain or loss present in dogs but not in any wolves. We found 190 and 150 post-domestication duplications and deletions, respectively. It has been suggested that gene deletions are more likely to be deleterious than duplications and therefore more likely to be purged by purifying selection. However, we did not find an enrichment in genes in the 190 regions with duplications in dogs compared to the whole set of 1,611 CNV regions (*P* = 0.519), while we found gene enrichment in the 150 regions with deletions (*P* < 0.001; Additional file 1: Figure S2) suggesting a potential relaxation of purifying selection in dogs. This is consistent with previous studies which have described a relative increase in the proportion of non synonymous substitutions in the dog genome, suggested to be the result of a relaxation of the purifying selection in dogs [8, 32]. This could be due to changes in the way of life of dogs and, specially, to the reduction of their effective population size compared to the population size of the ancestor species, the gray wolf, during domestication.

### Analysis of CNV breakpoints

**Figure 2:**
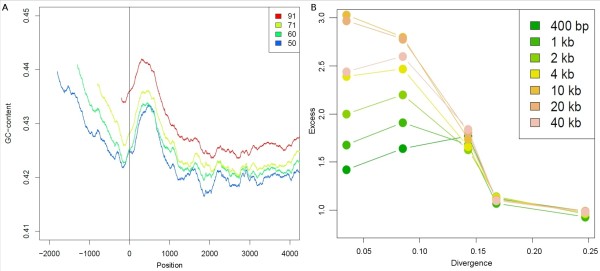
**CNV breakpoints composition analysis. A:** Base composition of CNV breakpoints. GC-content in 400 bp windows around the breakpoint recorded at the center of each window. Negative locations represent windows inside the CNVs and colors represent the proportion of CNVs with a size that can cover a window at a specific distance inside the CNVs. **B:** Enrichment of L1 repeats in CNV breakpoints. Observed to expected ratios of 5 classes of differentially diverged L1 repeats in CNV breakpoints. Colors represent the size of the CNV breakpoint.

Taking advantage of our higher aCGH resolution, we could define CNV breakpoints within 400 bp and analyze their nucleotide composition. GC-peaks were defined as 500 bp windows or greater centered in 10 kb windows with more than 50% increase in GC-content [21]. We found an even clearer enrichment of peaks of GC-high regions close to the breakpoints compared to previous results [21]. The enrichment rapidly decays outside breakpoints (steps of 400 bp) (Additional file 1: Figure S3). We next recorded the nucleotide fine-scale GC-content around the breakpoints in sliding windows of 400 bp (Figure 2). We found a small increase in GC-content about a kb outside the breakpoint, although there seemed to be a small local decrease in GC-content exactly at the breakpoint. However, our ability to locate the exact position of the breakpoints fluctuated over a few hundred bp given the probe distribution in the arrays (repeats, which are enriched in breakpoints are not covered by probes) and the CNV callers tended to have some uncertainty in the transition probes at the breakpoints. Assuming some uncertainties in the identification of the breakpoints, we still found local peaks around 1 kb from the breakpoint that could indicate some common motif, whereas the observed increase in GC-content within the CNVs could indicate the effects of biased gene conversion which increases GC-content in duplicated sequences.

We next searched for stretches of perfect homology between breakpoint pairs defined using the 400 bp windows. The longest stretch of perfect homology was recorded for paired breakpoints. The mean length was 10.9 bp. The pairs were then randomly redistributed on the same chromosome to evaluate statistical significance, with a mean of 9.2 bp using a Wilcoxon rank sum test. We found a small but significant increase in homology between breakpoint pairs compared to a random expectation, supporting NAHR as a main mechanism for formation of CNVs in canids. An even stronger effect is supported when increasing the breakpoint size to 2 kb to include the peculiar GC-pattern seen one kb away from the break; the homology stretch then increased to 22.8 bp vs. 14.2 bp expected by chance (*P* < 0.001, Wilcoxon rank sum test).

We finally searched for regions of overlap between breakpoint windows and repeats using the RepeatMasker Track. The repeat families Simple repeats and L1 repeats were enriched in breakpoint windows (*P* < 0.01, random resampling). When we divided L1 repeats according to their age, recent L1s were more enriched than older ones (Figure 2), although not as pronounced as previously observed (20). Statistical enrichment of L1 repeats varied with breakpoint size in a fashion where enrichment increased with window sizes up to 10 kb and slowly decreased with larger window sizes. Therefore CNV breakpoints tend to have young L1 repeats nearby, although they are not overlapping.

### Candidate CNV selected during dog domestication

**Figure 3:**
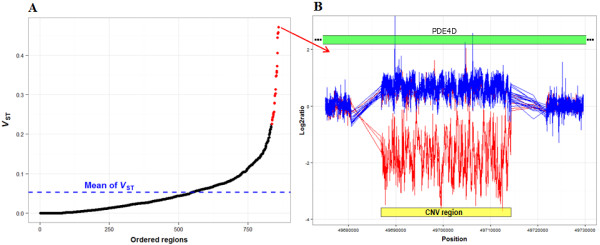
**Candidate CNVs selected during dog domestication. A:** Values of *V*_ST_ between dogs and wolves for the 860 regions (ordered by the *V*_ST_ value). In red, the 25 regions with highest *V*_ST_ values. **B:** Log2values of the region with highest *V*_ST_; dogs are represented in red. The CNV region (yellow) overlaps with the *PDE4D* gene (green).

Regions under selection early in dog domestication should be highly differentiated from those in the gray wolf, whereas regions selected during breed formation should show differentiation signals between dog breeds. Previous studies have focused on these later regions. To select the most differentiated regions between dog (including the dingo) and wild canids we calculated *V*_ST_ for each polymorphic region as previously described [30]. The distribution of *V*_ST_ showed that most of the regions (84.4%) had low (<0.1) *V*_ST_ (Figure 3), and the average *V*_ST_ (0.054) was lower than the *F*_ST_ obtained from SNP data [10]. Similarly, the estimates of *F*_ST_ for purebred dogs obtained from CNV data were also lower than the estimates obtained from SNP data [20]. This low estimate could be due to the smaller number of samples analyzed. However, we found regions with an estimate of *V*_ST_ several-fold higher than the average. For instance, within the 25 most differentiated regions, the lowest *V*_ST_ is 0.226 (>average *V*_ST_ + 2.5 SD) and the average *V*_ST_ is 0.319.

Of the 12 candidate genes in the most differentiated regions (Table 2), three genes are related to growth (*PDE4D, CRTC3* and *NEB*). The CNVs that include the *CRTC3* gene have higher copy number in dogs (with the exception of the dingo) than in gray wolves. It has been shown that *CRTC3*^−/−m^ ice maintained on a normal chow diet appear more insulin-sensitive than controls and also have 50% lower adipose tissue mass than control mice despite comparable physical activity [33]. Incidence of overweight and obesity in dogs exceeds 30%, and several breeds are predisposed to this heritable phenotype [34]. However, perhaps the most striking example of potential divergence in function is for the *PDE4D* gene (Figure 3). For this region, all wild canids present the same genotype (gain), whereas most of the studied dogs (Boxer, Beagle and Basenji) present losses. Mice that are deficient in this isoenzyme exhibit delayed growth with a 30-40% decrease in body weight at 1–2 weeks after birth [35]. Although growth rate returned to normal after 2 weeks, the weight of the adult mice remained lower than normal due to a decrease in muscle and bone mass and internal organ weight (with the exception of cortex and cerebellum) associated with a decrease in circulating insulin-like growth factor I (IGF1) levels. The *IGF1* gene is a strong genetic determinant of body size across mammals and a single *IGF1* allele is a major determinant of small size in dogs [1]. Consequently, CNVs near these genes may affect gene expression of this body size associated gene, or act as tag for sequence changes in the gene or its promoter that affect expression. In dogs, six genes explain ~50% of standard breed weight and it is hypothesized that these large-effect variants are superimposed on a subtler size-regulation system inherited from wolves [36]. Wolves vary substantially in size, with weights ranging from 16 to 60 kg in Europe alone [37]. On the other hand, PDE4 inhibitors also facilitate hippocampal long-term potentiation in addition to improving cognitive performance in multiple animal models and reverse memory impairments in genetic mouse models of human disorders [38]. In particular, *PDE4D*^−/−^ mice exhibited enhanced earlylong-term potentiation following multiple induction protocols [38].

**Table 2:** **List of top 25 most differentiated regions based on** ***V***_**ST**_ **between dog and wild canids**

***V***_ST_	Chr	Start	End	Gene
0.470	chr2	49,686,866	49,714,373	Phosphodiesterase 4D, cAMP-specific (*PDE4D*)
0.458	chr8	63,053,405	63,054,226	Echinoderm microtubule associated protein like 5(*EML5*)
0.455	chr9	20,104,325	20,942,683	
0.444	chr34	15,222,600	15,228,141	
0.405	chr3	56,422,480	56,425,666	CREB regulated transcription coactivator 3(*CRTC3*)
0.372	chr18	57,419,688	57,432,938	
0.360	chr10	11,064,284	11,065,425	
0.356	chr10	37,202,529	37,206,649	
0.348	chr6	40,916,498	40,919,469	Zinc finger protein 500 (*ZNF500*)
0.346	chr8	19,445,383	19,454,376	
0.313	chr28	35,223,479	35,250,497	Deleted in malignant brain tumors 1 (*DMBT1*)
0.303	chr26	15,768,680	15,778,843	
0.301	chr20	10,484,859	10,488,574	Solute carrier family 6 (neurotransmitter transporter), member 11(*SLC6A11*)
0.297	chr10	21,640,830	21,644,386	
0.279	chr7	35,432,582	35,437,565	Regulator of G-protein signaling 7 (*RGS7*)
0.278	chr22	34,304,138	34,305,222	EDNRB antisense RNA 1 (*EDNRB-AS1*)
0.257	chr28	16,096,188	16,106,945	
0.255	chr5	38,737,275	38,738,036	Dynein, axonemal, heavy chain 9 (*DNAH9*)
0.253	chr6	50,243,863	50,244,363	
0.249	chr26	31,903,962	31,981,330	
0.244	chr14	3,093,975	3,101,514	
0.243	chr8	11,405,495	11,411,624	
0.237	chr11	45,618,474	45,621,740	ELAV like neuron-specific RNA binding protein 2 (*ELAVL2*)
0.236	chr1	15,627,405	15,636,380	
0.226	chr26	34,271,837	34,692,378	Topoisomerase (DNA) III beta (*TOP3B*)*/*Nebulin (*NEB*)

Interestingly, among the 12 candidate genes, six other genes also are implicated in neurological function in other mammalian species (*EML5*, *ZNF500*, *SLC6A11*, *ELAVL2*, *RGS7* and *TOP3B*) [39–45]. The synaptic regulator *SLC6A11* is a particularly interesting candidate since human genetic studies indicate that a CNV including this gene is associated with autism spectrum disorders and schizophrenia [41]. One of the most unique behavioral traits of dogs relative to wolves is their social-communicative skills with humans. Domestic dogs are more skillful than chimpanzees and wolves at using human social clues to find hidden food in the object choice paradigm [46–48]. This trait likely enabled domestication and facilitated the rapid evolution of genes expressed in the brains of dogs [9, 49].

It is relevant that, among the 12 genes within highly differentiated CNV regions between dog and wolf 9 of them are related to two functions, typically associated with the process of domestication. However, further functional studies are needed to disentangle the complete role of these genes in the dog domestication process.

## Conclusions

In this study, we make use of previously reported CNVs in modern dog breeds to explore the evolutionary origin of these sites by using a novel panel of wolf-like canids.

This expanded dataset, combined with our custom-designed higher density array, allowing us to determine the ancestral state and polarize the process of CNV formation in dogs. We identified some candidate genes within CNV regions that are highly differentiated between dogs and wolves, which provide insights into the role of structural variation in the process of dog domestication and in diversification of phenotypes observed among dog breeds. In general our results add significantly to resolution of structural variation and breakpoints in canids. However, ascertainment bias is a problem for the interpretation of CNV patterns in wild canids and analyses of CNVs based on whole genome sequencing will be highly beneficial to evaluate the evolution and impact of structural variability in the process of domestication.

## Methods

### DNA samples

A female Boxer (distinct from Tasha, used by Nicholas et al. [19, 20] and whose genome was sequenced [14]) was used as reference in all the aCGH hybridizations. The samples used for the aCGH experiments corresponded to four purebred dogs (from four breeds: Boxer, Dachshund, Beagle and Basenji), one Dingo, 15 gray wolves, one red wolf, one coyote and one golden jackal. The origin of these wolf samples covers a large geographic range, including European, American and Asian populations (Table 1). All wolf samples derive from animals killed or found dead for reasons other than this research and deposited in scientific collections. Dog samples derive from veterinary clinics and were obtained with the permission of the owner. A total of two self-self hybridizations were done using a Boxer and an Iranian wolf. DNA quality of all samples was assessed by taking OD260/280 and OD260/230 readings using a nanospectrometer and agarose gel electrophoresis. Hybridizations of genomic DNA to NimbleGen aCGH chip were performed in the Genomics Core Facility of the Centre for Genomic Regulation (CRG) in Barcelona (Spain).

### Array design

A NimbleGen aCGH chip was designed to sample the same regions covered in [20], but with higher density. Specifically, the mean probe space varied depending on the length of the tiled region. For regions smaller than 100 kb (93% of the regions), the mean probe space was 50 bp; for regions between 100 and 300 kb (5%), probes were separated by 150 bp on average and finally, for regions longer than 300 kb (2%), mean probe spacing was 1 kb. Furthermore, 42 putative control regions were included in the chip. Overall, the chip contains 598,733 probes with an average probe spacing of 157 bp.

### Validation of CNV regions by qPCR

We performed qPCR on 4 dogs (included the Boxer), 3 wolves, 1 coyote and 2 jackals from 3 CNV regions that involve *PDE4D*, *CRTC3* and *SLC6A11* genes, all of them present in Table 2.

Estimation of copy number was performed using a Multiplex TaqMan assays. Each duplex reaction contained TaqMan probes and primers to amplify C7orf28B [6], which is known to exist in two copies in a canid genome (900 nM of forward and reverse primers, 250 nM VIC and TAMRA labeled probe, Applied Biosystems), and the TaqMan probes and primers (Additional file 1: Table S3) used to amplify the test regions (300 nM of forward and reverse primers, 250 nM FAM labeled MGB probe, Applied Biosystems). Amplicons were done in genomic DNA under the following conditions: one cycle at 50°C for 2 min, one cycle at 95°C for 10 min and 40 cycles at 95°C for 15 sec, 55°C for 30 sec and 72°C for 30 sec. Three replicates were performed for each sample.

### CNV genotyping

We first identified CNV regions in each sample using two methods: a Reversible Jump hidden Markov Model implemented in the software RJaCGH [26] and the procedure described in [21]. For the first method, we required an average posterior probability of the probes in the putative CNV greater than 0.60 if the segment consisted of at least 50 probes and greater than 0.75 if the segment had between 30 and 49 probes. We discarded segments with less than 30 probes. Then, for each sample we joined CNV regions if they fulfilled at least one of the following conditions: they were less than 3kb apart from each other or the region between them had more than 80% repeats or gaps (downloaded from the UCSC Table Browser). Next, overlapping CNV regions were merged across all the samples in order to define a set of 860 regions that were used for the genotyping step.

In the genotyping step, we genotyped each sample in the 860 regions previously identified, requiring an average log_2_value of the region equal to the median ± 1.5 * standard deviation of all log_2_values of the chip.

### Statistical and population genetics analysis

Genotypes were simplified into 3 categories: equal copy, gain and loss, and allele frequencies for each category were estimated using a simple EM algorithm. These allele frequencies were used to calculate expected heterozygosity in each of the 860 regions for dogs and wolves as He =1- (p^2^ + q^2^ + r^2^), where p, q, and r indicate the frequencies of samples carrying normal copy, gains, and losses, respectively. Furthermore, we computed *V*_ST_ for each CNV region as: *V*_ST_ = (*V*_T_ - *V*_S_)/*V*_T_, where *V*_T_ is the variance in log_2_ ratios among all unrelated individuals and *V*_S_ is the average variance within each population, weighted for population size.

### Candidate genes

We downloaded a complete list of all canine genes from Ensemble, which comprised 24,580 genes in CanFam3.1 coordinates.

In order to determine the genes that a given set of CNV regions contain or overlapped, we first used liftOver (http://genome.ucsc.edu/cgi-bin/hgLiftOver) to map the coordinates of the regions of interest to CanFam3.1 coordinates. Then, we intersected those coordinates with the gene list.

The list of genes was analyzed with PANTHER (Protein ANalysisTHrough Evolutionary Relationships) [50] using default options. PANTHER provides a functional analysis combining GO.

Next, to investigate whether a given set of CNV regions was significantly enriched or depleted in genes, 1,000 sets with the same number and length of regions were simulated across either the 1,611 regions analyzed or the 754 non dog specific regions. The number of genes for each of the simulated sets was calculated, and compared with the original set to obtain statistical significance.

### Analyses on the breakpoints

Breakpoints were defined as windows of 400 bp, the smallest size of any detected CNV, surrounding the inferred breakpoint position to account for the imprecision in determining the exact location.

Peaks of elevated GC-content were defined as in [21], with a 500 bp peak discovery window centered in a 10 kb background window. To record peaks, these two windows were simultaneously slid along the genome to detect increased levels of GC-content of 50% in the peak window relative to the background window.

Analyses of enrichment and overlap between genomic features were done chromosome-wise by repeatedly and randomly redistributing the regions to estimate sample means to infer statistical significance. The two breakpoints of a CNV were kept at the same distance from each other during the process.

Repeat locations came from the RepeatMasker track of the UCSC genome browser (genome.ucsc.edu). L1 repeats were divided according to their age (origin from *Canisfamiliaris*, *Canis*, Canidae, Carnivora, older Mammalia/Eutheria) using Repbase (http://www.girinst.org/repbase/).

## Electronic supplementary material

Additional file 1: **File containing Table S1, and S3, Figure S1, S2, S3 and their legends.** (PDF 2 MB)

Additional file 2: **Excel file containing Table S2.** (XLSX 91 KB)
