# Supplementary material for: Analysis of structural diversity in wolf-like canids reveals post-domestication variants
Source: BMC Genomics. 2014 Jun 12;15(1):465. doi: 10.1186/1471-2164-15-465 (PMC4070573; doi:10.1186/1471-2164-15-465)

Table S1. List of 1611 CNVs regions analyzed. Location in the canine genome (in CanFam2) and the reference where the region was identified as CNV.

Id	Location	Nicholas et al 2009	Nicholas et al 2011	Chen et al 2009	Nicholas et al 2011b*	Control
1	chr1:7659258-7685115	1	0	0	1	0
2	chr1:15383000-15433000	0	0	0	1	1
3	chr1:15622923-15641362	0	0	0	1	0
4	chr1:17028737-17049272	0	0	0	1	0
5	chr1:19280766-19389071	0	0	1	0	0
6	chr1:26658693-26672654	0	0	0	1	0
7	chr1:27081367-27101856	0	0	0	1	0
8	chr1:27689981-27728640	0	1	0	1	0
9	chr1:28637185-28651382	0	0	0	1	0
10	chr1:28741708-28774713	0	1	0	1	0
11	chr1:30845204-30861811	0	0	0	1	0
12	chr1:32761783-32800255	0	1	0	1	0
13	chr1:33574200-33590460	0	0	0	1	0
14	chr1:43628850-43673566	0	1	0	1	0
15	chr1:44540004-44555203	0	0	0	1	0
16	chr1:46735977-46756513	0	0	0	1	0
17	chr1:53314209-53332483	0	0	0	1	0
18	chr1:58374583-58388605	0	0	0	1	0
19	chr1:59729895-59744110	0	0	0	1	0
20	chr1:59932647-59950197	0	0	0	1	0
21	chr1:59957851-59977386	0	0	0	1	0
22	chr1:60496517-60512605	0	0	0	1	0
23	chr1:61731954-61751501	0	0	0	1	0
24	chr1:62878468-62901251	0	0	0	1	0
25	chr1:64182758-64197008	0	0	0	1	0
26	chr1:66101746-66118074	0	0	0	1	0
27	chr1:66799863-66856988	0	1	0	1	0
28	chr1:68993035-69013347	0	0	0	1	0
29	chr1:69337580-69355902	0	0	0	1	0
30	chr1:71791767-71815413	0	0	0	1	0
31	chr1:72605352-72629541	1	0	0	0	0
32	chr1:75143671-75165349	0	0	0	1	0
33	chr1:75721894-75743567	0	0	0	1	0
34	chr1:77236298-77256256	0	0	0	1	0
35	chr1:78056944-78176123	1	0	0	1	0
36	chr1:78214715-78251568	1	0	0	0	0
37	chr1:78266301-78283886	0	0	0	1	0
38	chr1:80539028-80556301	0	0	0	1	0
39	chr1:82564821-82580119	0	0	0	1	0
40	chr1:84602417-84619744	0	0	0	1	0
41	chr1:87468936-87483131	0	0	0	1	0
42	chr1:91402045-91420264	0	0	0	1	0
43	chr1:94251848-94268251	0	0	0	1	0
44	chr1:95904579-95918782	0	0	0	1	0
45	chr1:97837108-97862211	0	0	0	1	0
46	chr1:99031985-99064045	0	0	0	1	0
47	chr1:100206591-100224997	0	0	0	1	0
48	chr1:104590912-104613831	0	1	0	1	0
49	chr1:104811883-104828323	0	0	0	1	0

50	chr1:104914544-105079782	0	1	0	0	0
51	chr1:105196217-105226299	0	0	0	1	0
52	chr1:105636540-105667700	0	0	0	1	0
53	chr1:105731316-105751532	0	0	0	1	0
54	chr1:105809893-105848142	0	1	0	1	0
55	chr1:105928012-105942317	0	0	0	1	0
56	chr1:106071301-106093839	0	0	0	1	0
57	chr1:106151996-106173446	0	0	0	1	0
58	chr1:106217333-106231354	0	0	0	1	0
59	chr1:106975904-107149173	1	0	0	0	0
60	chr1:107159760-107442965	1	1	0	1	0
61	chr1:107459055-107477741	0	0	0	1	0
62	chr1:107558390-107589442	0	1	0	1	0
63	chr1:107719005-107733181	0	0	0	1	0
64	chr1:108107502-108126036	1	0	0	0	0
65	chr1:108154205-108172475	1	0	0	0	0
66	chr1:108881250-108919537	1	0	0	1	0
67	chr1:109797183-109815579	0	0	0	1	0
68	chr1:110233510-110247811	0	0	0	1	0
69	chr1:110880346-110941958	1	0	0	0	0
70	chr1:111613968-111642803	0	1	0	1	0
71	chr1:111959282-111977077	0	0	0	1	0
72	chr1:112987649-113007101	0	0	0	1	0
73	chr1:113340933-113364404	0	0	0	1	0
74	chr1:114099203-114115596	0	0	0	1	0
75	chr1:114631409-114718134	1	1	0	1	0
76	chr1:114912020-114927292	0	0	0	1	0
77	chr1:115156798-115178662	0	0	0	1	0
78	chr1:115208422-115234438	0	0	0	1	0
79	chr1:115260471-115329604	0	0	0	1	0
80	chr1:115412042-115472278	1	1	0	1	0
81	chr1:115971283-115989519	0	0	0	1	0
82	chr1:117010703-117024841	0	0	0	1	0
83	chr1:119398855-119423536	0	0	0	1	0
84	chr1:120002222-120021539	0	0	0	1	0
85	chr1:120216872-120236307	0	0	0	1	0
86	chr1:121253029-121286091	0	0	0	1	0
87	chr1:121367360-121396431	0	0	0	1	0
88	chr1:121580654-121596996	0	0	0	1	0
89	chr1:121812079-121830453	0	0	0	1	0
90	chr1:123351166-123371826	0	0	0	1	0
91	chr1:123462572-123486226	0	0	0	1	0
92	chr1:123700987-123717466	0	0	0	1	0
93	chr1:124611126-124629338	0	0	0	1	0
94	chr1:125485244-125500485	0	0	0	1	0
95	chr2:6937752-7344511	1	1	0	1	0
96	chr2:7465432-8002404	1	1	0	1	0
97	chr2:8067467-8671097	1	1	0	1	0
98	chr2:8690906-8897261	1	1	0	1	0
99	chr2:9195149-9215457	1	0	0	0	0
100	chr2:9250664-9270777	1	0	0	0	0
101	chr2:9289070-9341263	1	0	0	1	0

102	chr2:9346941-9664062	1	0	0	1	0
103	chr2:10668800-10688296	0	0	0	1	0
104	chr2:10832545-10847840	0	0	0	1	0
105	chr2:12859189-12879789	0	1	0	1	0
106	chr2:16344231-16383006	1	0	0	1	0
107	chr2:16609941-16682417	1	0	0	1	0
108	chr2:17384236-17409701	0	1	0	1	0
109	chr2:19322578-19337960	0	0	0	1	0
110	chr2:20393148-20442004	1	1	0	1	0
111	chr2:20533007-20547177	0	0	0	1	0
112	chr2:20735044-20765084	1	1	0	1	0
113	chr2:20879135-20963539	0	1	0	1	0
114	chr2:21064053-21272262	1	1	0	1	0
115	chr2:24576426-24590515	0	0	0	1	0
116	chr2:26022033-26099460	1	1	1	1	0
117	chr2:26597057-26618101	0	1	0	1	0
118	chr2:32087247-32106773	0	0	0	1	0
119	chr2:33001971-33020446	0	0	0	1	0
120	chr2:38745009-38783072	1	1	0	1	0
121	chr2:38801450-38848052	0	1	0	1	0
122	chr2:39084100-39107277	1	0	0	0	0
123	chr2:39215152-39234408	0	1	0	1	0
124	chr2:39808519-39862903	0	0	1	0	0
125	chr2:45313786-45331432	0	0	0	1	0
126	chr2:48673677-48690945	0	1	0	1	0
127	chr2:49389395-49403677	0	0	0	1	0
128	chr2:49675101-49729636	0	1	1	1	0
129	chr2:50269550-50369550	0	0	0	0	1
130	chr2:54746452-54763754	0	0	0	1	0
131	chr2:55923786-55946502	0	1	0	1	0
132	chr2:59176253-59191703	0	0	0	1	0
133	chr2:60302292-60596222	1	1	0	0	0
134	chr2:60748726-60770490	1	0	0	0	0
135	chr2:63717840-63733934	0	0	0	1	0
136	chr2:65677798-65693102	0	0	0	1	0
137	chr2:66666072-66683567	0	0	0	1	0
138	chr2:67315587-67329803	0	0	0	1	0
139	chr2:68148519-68186928	1	0	0	0	0
140	chr2:68406274-68424835	0	0	0	1	0
141	chr2:70859864-70875179	0	0	0	1	0
142	chr2:72207268-72223660	0	0	0	1	0
143	chr2:72345433-72360746	0	0	0	1	0
144	chr2:73666480-73680700	0	0	0	1	0
145	chr2:74086211-74102310	0	0	0	1	0
146	chr2:76152533-76167744	0	0	0	1	0
147	chr2:79579670-79593973	0	0	0	1	0
148	chr2:79706845-79725195	0	0	0	1	0
149	chr2:80727050-80756805	0	1	0	0	0
150	chr2:82686402-82700522	0	0	0	1	0
151	chr2:82896397-82913662	0	0	0	1	0
152	chr2:83156387-83175916	0	0	0	1	0
153	chr2:83389857-83406141	0	0	0	1	0

154	chr2:83557615-83575017	0	0	0	1	0
155	chr2:84970506-84989964	0	0	0	1	0
156	chr2:85419745-85436022	0	0	0	1	0
157	chr2:85844001-85871057	1	0	0	0	0
158	chr2:86045773-86084071	0	0	0	1	0
159	chr2:86444251-86460556	0	0	0	1	0
160	chr2:86689359-86716448	1	1	0	1	0
161	chr2:86809122-87032410	1	1	1	1	0
162	chr3:3035595-3054185	1	0	0	0	0
163	chr3:11491027-11512259	1	0	0	0	0
164	chr3:15527958-15543350	0	0	0	1	0
165	chr3:15963943-15980085	0	0	0	1	0
166	chr3:20408514-20425866	0	0	0	1	0
167	chr3:20755751-20775177	0	0	0	1	0
168	chr3:24344541-24363999	0	1	0	1	0
169	chr3:25552440-25566633	0	0	0	1	0
170	chr3:26051916-26151916	0	0	0	1	1
171	chr3:26906990-26922200	0	0	0	1	0
172	chr3:30551123-30569520	0	0	0	1	0
173	chr3:30625661-30645222	0	0	0	1	0
174	chr3:33558565-33573230	0	0	0	1	0
175	chr3:34147139-34446301	1	1	0	1	0
176	chr3:34480078-34505701	0	0	0	1	0
177	chr3:36091319-36106467	0	0	0	1	0
178	chr3:36499571-36517160	0	0	0	1	0
179	chr3:38570956-38600036	1	0	0	0	0
180	chr3:39327351-39381771	0	1	0	1	0
181	chr3:48273124-48373124	0	0	0	0	1
182	chr3:51348214-51369154	0	0	0	1	0
183	chr3:52417499-52437033	0	0	0	1	0
184	chr3:54220968-54235231	0	0	0	1	0
185	chr3:54487175-54504622	0	0	0	1	0
186	chr3:54525671-54542096	0	0	0	1	0
187	chr3:54667835-54685056	0	0	0	1	0
188	chr3:54738262-54753502	0	0	0	1	0
189	chr3:55730442-55752171	0	0	0	1	0
190	chr3:56411806-56437854	0	0	0	1	0
191	chr3:57859183-57875499	0	0	0	1	0
192	chr3:59398705-59413009	0	0	0	1	0
193	chr3:60003863-60018016	0	0	0	1	0
194	chr3:60430431-60451911	0	0	0	1	0
195	chr3:61162303-61180735	0	0	0	1	0
196	chr3:64324102-64342360	0	0	0	1	0
197	chr3:69705871-69724320	0	0	0	1	0
198	chr3:70576673-70593025	0	0	0	1	0
199	chr3:71156303-71175694	0	0	0	1	0
200	chr3:71427491-71464942	1	1	0	1	0
201	chr3:71925082-71944747	0	0	0	1	0
202	chr3:71956663-71981542	0	0	0	1	0
203	chr3:72050603-72075362	0	0	0	1	0
204	chr3:74227292-74354522	0	1	0	0	0
205	chr3:74637875-74653037	0	0	0	1	0

206	chr3:74943417-74966259	0	0	0	1	0
207	chr3:75330892-75347103	0	1	0	1	0
208	chr3:76343002-76374076	0	1	0	1	0
209	chr3:77052929-77074565	0	0	0	1	0
210	chr3:84938901-84954398	0	0	0	1	0
211	chr3:89639311-89654708	0	0	0	1	0
212	chr3:90017648-90032351	0	0	0	1	0
213	chr3:92398093-92419610	0	1	0	1	0
214	chr3:93638750-93658328	0	1	0	1	0
215	chr3:94380307-94402996	0	0	0	1	0
216	chr4:3098903-3149905	0	1	0	1	0
217	chr4:4093733-4115163	0	0	0	1	0
218	chr4:5340132-5355410	0	0	0	1	0
219	chr4:6012868-6033281	0	1	0	1	0
220	chr4:11019949-11035203	0	0	0	1	0
221	chr4:13099421-13125085	0	1	0	1	0
222	chr4:13457039-13471935	0	1	0	1	0
223	chr4:15272536-15287761	0	0	0	1	0
224	chr4:18914276-18948422	0	1	0	1	0
225	chr4:19801539-19827525	0	1	0	1	0
226	chr4:21137148-21200806	0	0	1	0	0
227	chr4:26270379-26284584	0	0	0	1	0
228	chr4:30495780-30595780	1	0	0	0	1
229	chr4:31146500-31168045	0	0	0	1	0
230	chr4:32780995-32807116	0	0	0	1	0
231	chr4:33835234-33851601	0	0	0	1	0
232	chr4:35062142-35078546	0	0	0	1	0
233	chr4:35270211-35284595	0	0	0	1	0
234	chr4:36165454-36180610	0	0	0	1	0
235	chr4:37007720-37028160	0	0	0	1	0
236	chr4:39775616-39792985	0	1	0	1	0
237	chr4:40101162-40119640	0	1	0	1	0
238	chr4:40291238-40355985	0	1	0	1	0
239	chr4:41170228-41187667	0	0	0	1	0
240	chr4:44002925-44018103	0	0	0	1	0
241	chr4:44692595-44709701	0	0	0	1	0
242	chr4:51004846-51018996	0	0	0	1	0
243	chr4:51841860-51861494	0	0	0	1	0
244	chr4:53488500-53588500	0	0	0	0	1
245	chr4:57679302-57743303	0	0	1	0	0
246	chr4:59827159-59846670	0	1	0	1	0
247	chr4:61441171-61463512	1	0	0	1	0
248	chr4:61615878-61632165	0	0	0	1	0
249	chr4:62280725-62301200	0	0	0	1	0
250	chr4:63542354-63627370	0	0	1	1	0
251	chr4:65733662-65751092	0	0	0	1	0
252	chr4:66066703-66086199	0	1	0	1	0
253	chr4:66243787-66259004	0	0	0	1	0
254	chr4:67702004-67718239	0	0	0	1	0
255	chr4:69745893-69763241	0	0	0	1	0
256	chr4:70688966-70720060	0	1	0	1	0
257	chr4:71606943-71622110	0	0	0	1	0

258	chr4:73578094-73595558	0	0	0	1	0
259	chr4:73773301-73790457	0	0	0	1	0
260	chr4:77200642-77214817	0	0	0	1	0
261	chr4:77649573-77679587	0	1	0	1	0
262	chr4:78667758-78681984	0	0	0	1	0
263	chr4:80044081-80058146	0	0	0	1	0
264	chr4:80704204-80719407	0	0	0	1	0
265	chr4:81111133-81130524	0	0	0	1	0
266	chr4:85396521-85415056	0	0	0	1	0
267	chr4:86654851-86676432	0	0	0	1	0
268	chr4:87620723-87637929	0	0	0	1	0
269	chr4:89280530-89295926	0	0	0	1	0
270	chr4:89522399-89539561	0	0	0	1	0
271	chr4:89716460-89732654	0	0	0	1	0
272	chr4:90657700-90680209	0	0	0	1	0
273	chr4:91215881-91229914	0	0	0	1	0
274	chr5:2996081-3074982	1	1	0	1	0
275	chr5:4300227-4314579	0	1	0	0	0
276	chr5:11240988-11259250	0	0	0	1	0
277	chr5:11734973-11750705	0	0	0	1	0
278	chr5:13118625-13137250	0	0	0	1	0
279	chr5:14549542-14570136	0	0	0	1	0
280	chr5:15858158-15872445	0	0	0	1	0
281	chr5:16383332-16399634	0	0	0	1	0
282	chr5:18950931-18969283	0	0	0	1	0
283	chr5:25390148-25406345	0	0	0	1	0
284	chr5:26086151-26102433	0	0	0	1	0
285	chr5:28879694-28901980	0	1	0	1	0
286	chr5:31040478-31062529	0	0	0	1	0
287	chr5:31095735-31111975	0	0	0	1	0
288	chr5:32833617-32856245	0	0	0	1	0
289	chr5:33061557-33083734	1	0	0	0	0
290	chr5:33098635-33112663	0	0	0	1	0
291	chr5:34093139-34175180	0	0	1	0	0
292	chr5:38499340-38516797	0	0	0	1	0
293	chr5:38726264-38742753	0	0	0	1	0
294	chr5:38843379-38857542	0	0	0	1	0
295	chr5:40574428-40589826	0	0	0	1	0
296	chr5:41795848-41809946	0	0	0	1	0
297	chr5:42030918-42052339	0	0	0	1	0
298	chr5:42256289-42275914	0	1	0	1	0
299	chr5:44115860-44137322	0	0	0	1	0
300	chr5:44956842-44979505	0	1	0	0	0
301	chr5:46335506-46353958	0	0	0	1	0
302	chr5:47601449-47615750	0	0	0	1	0
303	chr5:49764928-49786381	0	0	0	1	0
304	chr5:49818962-49838363	0	1	0	1	0
305	chr5:52878803-52892905	0	0	0	1	0
306	chr5:53084589-53098882	0	1	0	1	0
307	chr5:57216555-57230850	0	0	0	1	0
308	chr5:59554851-59576500	0	0	0	1	0
309	chr5:61424520-61524520	0	0	0	0	1

310	chr5:61711614-61727986	0	0	0	1	0
311	chr5:62369977-62385237	0	0	0	1	0
312	chr5:68858135-68877436	0	0	0	1	0
313	chr5:70246459-70260744	0	0	0	1	0
314	chr5:72643311-72658594	0	0	0	1	0
315	chr5:75358999-75374210	0	0	0	1	0
316	chr5:78662688-78677966	0	0	0	1	0
317	chr5:81143006-81475905	0	1	1	1	0
318	chr5:83273029-83288164	0	1	0	1	0
319	chr5:83805336-83819678	0	0	0	1	0
320	chr5:84060003-84160003	0	0	0	0	1
321	chr5:84796587-84816756	0	0	0	1	0
322	chr5:86179277-86212449	0	0	0	1	0
323	chr5:86567046-86583391	0	0	0	1	0
324	chr5:87903603-87917871	0	0	0	1	0
325	chr5:88633863-88657358	0	0	0	1	0
326	chr5:90973772-90994346	0	0	0	1	0
327	chr5:91187041-91201280	0	0	0	1	0
328	chr5:91354516-91374904	0	0	0	1	0
329	chr6:7394764-7412257	0	0	0	1	0
330	chr6:8284327-8298570	0	0	0	1	0
331	chr6:11634410-11650018	0	0	0	1	0
332	chr6:11795814-11829236	0	1	0	1	0
333	chr6:12022963-12040287	0	0	0	1	0
334	chr6:12736643-12771611	1	0	0	0	0
335	chr6:12785979-12929586	1	1	1	1	0
336	chr6:14360605-14377929	0	0	0	1	0
337	chr6:15054478-15093875	1	1	0	1	0
338	chr6:15175203-15190411	0	0	0	1	0
339	chr6:17462041-17476115	0	0	0	1	0
340	chr6:17543174-17605613	0	0	0	1	0
341	chr6:19046345-19065819	0	0	0	1	0
342	chr6:19730239-19748092	0	1	0	1	0
343	chr6:19834036-19894972	1	0	0	0	0
344	chr6:20078345-20093479	0	0	0	1	0
345	chr6:21876547-21890551	0	0	0	1	0
346	chr6:29125009-29155013	1	0	0	0	0
347	chr6:29893060-29907298	0	0	0	1	0
348	chr6:31207016-31222451	0	1	0	1	0
349	chr6:31899151-31916281	0	0	0	1	0
350	chr6:33331518-33348710	0	0	0	1	0
351	chr6:37666223-37688817	0	1	0	1	0
352	chr6:38240447-38293585	0	1	0	1	0
353	chr6:39557092-39573470	0	0	0	1	0
354	chr6:40876217-40974223	1	0	0	0	0
355	chr6:43552234-43582804	1	0	0	0	0
356	chr6:43692666-43808091	1	0	1	1	0
357	chr6:46117146-46149042	1	1	0	1	0
358	chr6:47537888-47559526	0	1	0	1	0
359	chr6:48247942-50166652	1	1	1	1	0
360	chr6:50232414-50250053	0	0	0	1	0
361	chr6:51950115-51971829	0	0	0	1	0

362	chr6:53687008-53701073	0	0	0	1	0
363	chr6:55915769-55940485	0	1	0	1	0
364	chr6:60888058-60907036	0	0	0	1	0
365	chr6:61814200-61829442	0	0	0	1	0
366	chr6:62774801-62788980	0	0	0	1	0
367	chr6:64382458-64399653	0	0	0	1	0
368	chr6:67213485-67230771	0	0	0	1	0
369	chr6:67345888-67445888	0	0	0	0	1
370	chr6:67925327-67943811	0	1	0	1	0
371	chr6:69221891-69239221	0	0	0	1	0
372	chr6:71263479-71281877	0	0	0	1	0
373	chr6:71519460-71534941	0	0	0	1	0
374	chr6:72794429-72809552	0	0	0	1	0
375	chr6:73429809-73447218	0	0	0	1	0
376	chr6:73620652-73635941	0	0	0	1	0
377	chr6:73728825-73743175	0	0	0	1	0
378	chr6:74131783-74159556	0	1	0	1	0
379	chr6:74824389-74841659	0	0	0	1	0
380	chr6:75460915-75475081	0	0	0	1	0
381	chr6:76701751-76716122	0	0	0	1	0
382	chr6:78363491-78379552	0	0	0	1	0
383	chr6:79261889-79279277	0	0	0	1	0
384	chr6:79296620-79311884	0	0	0	1	0
385	chr6:79573233-79589548	0	0	0	1	0
386	chr6:79794305-79812698	0	0	0	1	0
387	chr7:9834685-9850856	0	0	0	1	0
388	chr7:14389504-14406941	0	0	0	1	0
389	chr7:17520754-17544424	0	0	0	1	0
390	chr7:18395857-18428705	0	1	0	1	0
391	chr7:23616250-23630571	0	0	0	1	0
392	chr7:24668013-24768013	0	0	0	0	1
393	chr7:27836341-27853656	0	0	0	1	0
394	chr7:30130624-30151069	0	1	0	1	0
395	chr7:30203047-30222582	0	1	0	1	0
396	chr7:33770591-33786905	0	0	0	1	0
397	chr7:34887012-34907587	0	0	0	1	0
398	chr7:35187243-35206591	0	0	0	1	0
399	chr7:35427773-35441875	0	0	0	1	0
400	chr7:42097650-42113901	0	0	0	1	0
401	chr7:42264801-42280974	0	0	0	1	0
402	chr7:42785667-42802009	0	0	0	1	0
403	chr7:43223795-43239117	0	0	0	1	0
404	chr7:43381751-43401320	0	1	0	1	0
405	chr7:43711751-43776141	1	1	0	1	0
406	chr7:43891291-43905466	0	0	0	1	0
407	chr7:44215060-44234507	0	0	0	1	0
408	chr7:46759465-46785299	0	1	0	1	0
409	chr7:47881499-47904198	0	0	0	1	0
410	chr7:49195585-49215208	0	0	0	1	0
411	chr7:50284442-50298663	0	0	0	1	0
412	chr7:53988415-54088415	0	0	0	0	1
413	chr7:57302673-57322130	0	0	0	1	0

414	chr7:58285371-58300725	0	0	0	1	0
415	chr7:62333189-62354775	0	1	0	1	0
416	chr7:63045588-63070105	0	0	0	1	0
417	chr7:64062188-64080651	0	0	0	1	0
418	chr7:65062670-65080961	0	0	0	1	0
419	chr7:70556322-70584206	0	1	0	1	0
420	chr7:71544449-71565631	0	1	0	1	0
421	chr7:72200401-72214579	0	0	0	1	0
422	chr7:74216313-74280062	0	1	0	1	0
423	chr7:76844681-76858777	0	0	0	1	0
424	chr7:77548998-77563162	0	0	0	1	0
425	chr7:77950247-77968413	0	1	0	1	0
426	chr7:79735722-79790353	0	0	1	0	0
427	chr7:82068403-82085556	0	0	0	1	0
428	chr7:82404092-82432964	0	1	0	1	0
429	chr7:83247599-83263842	0	0	0	1	0
430	chr7:83956919-83992106	1	0	0	0	0
431	chr8:3052358-3094061	1	0	0	1	0
432	chr8:3143456-3162881	0	1	0	1	0
433	chr8:3466112-3752747	0	1	0	1	0
434	chr8:4554651-4662058	0	1	0	1	0
435	chr8:5239689-5439539	1	0	0	0	0
436	chr8:6632387-6672858	0	1	0	1	0
437	chr8:9320910-9334977	0	0	0	1	0
438	chr8:11348806-11370805	0	1	0	1	0
439	chr8:11399935-11426053	0	1	0	1	0
440	chr8:13303548-13320664	0	1	0	1	0
441	chr8:16283803-16298049	0	0	0	1	0
442	chr8:16423354-16441573	0	0	0	1	0
443	chr8:19440384-19463274	0	1	0	1	0
444	chr8:19487676-19537676	0	0	0	0	1
445	chr8:21421310-21442757	0	1	0	1	0
446	chr8:22552030-22729253	0	1	0	0	0
447	chr8:23361271-23405915	0	1	0	1	0
448	chr8:27514775-27533312	0	0	0	1	0
449	chr8:31264442-31280561	0	0	0	1	0
450	chr8:36757467-36783376	0	1	0	1	0
451	chr8:41063788-41080117	0	1	0	1	0
452	chr8:47137418-47160360	0	1	0	1	0
453	chr8:47183377-48074839	1	1	0	1	0
454	chr8:53746856-53785621	0	1	0	1	0
455	chr8:57839964-57857497	0	0	0	1	0
456	chr8:57987301-58002411	0	0	0	1	0
457	chr8:59108084-59139186	1	1	0	1	0
458	chr8:62740272-62755555	0	0	0	1	0
459	chr8:63041880-63058577	0	0	0	1	0
460	chr8:63746451-63763641	0	0	0	1	0
461	chr8:64387696-64407026	0	0	0	1	0
462	chr8:64818315-64837622	0	0	0	1	0
463	chr8:65955819-65975359	0	0	0	1	0
464	chr8:66624618-66651236	0	0	0	1	0
465	chr8:66938822-66955276	0	0	0	1	0

466	chr8:68150016-68168360	0	0	0	1	0
467	chr8:68636237-68658815	0	0	0	1	0
468	chr8:68683678-68702955	0	0	0	1	0
469	chr8:68803360-68826204	0	0	0	1	0
470	chr8:69369915-69390420	0	0	0	1	0
471	chr8:69812210-69827380	0	0	0	1	0
472	chr8:72846503-72860806	0	0	0	1	0
473	chr8:74948962-74971824	0	0	0	1	0
474	chr8:76089443-76156150	1	0	0	0	0
475	chr8:76220436-76269535	1	0	0	1	0
476	chr8:76331651-77310517	1	1	1	1	0
477	chr9:6917096-6933416	0	0	0	1	0
478	chr9:9044602-9064918	0	1	0	0	0
479	chr9:10409074-10550071	1	1	0	1	0
480	chr9:10694484-11014094	1	1	0	1	0
481	chr9:11611157-12063771	1	1	1	1	0
482	chr9:13345206-13426806	1	1	0	1	0
483	chr9:13448777-13516710	1	1	1	1	0
484	chr9:15703271-15718520	0	0	0	1	0
485	chr9:17655236-17673443	0	1	0	1	0
486	chr9:19761852-21600512	1	1	1	1	0
487	chr9:22410910-22432607	0	1	0	0	0
488	chr9:22573547-22589779	0	0	0	1	0
489	chr9:23160606-23224386	1	1	0	1	0
490	chr9:24814531-24893355	1	1	0	1	0
491	chr9:26725593-26745085	0	0	0	1	0
492	chr9:26948549-26972072	0	0	0	1	0
493	chr9:28436048-28452307	0	0	0	1	0
494	chr9:34877130-34922451	1	1	0	1	0
495	chr9:36149125-36169736	0	0	0	1	0
496	chr9:42296347-42323187	0	1	0	1	0
497	chr9:42565268-42582619	0	0	0	1	0
498	chr9:43481097-43495939	0	0	0	1	0
499	chr9:45917465-45931464	0	1	0	0	0
500	chr9:49450214-49465381	0	0	0	1	0
501	chr9:50567380-50584664	0	0	0	1	0
502	chr9:50630089-50657754	1	1	0	1	0
503	chr9:53097716-53112956	0	0	0	1	0
504	chr9:53167014-53181252	0	0	0	1	0
505	chr9:55675258-55813750	0	1	0	0	0
506	chr9:56138796-56153916	0	0	0	1	0
507	chr10:5522341-5537592	0	0	0	1	0
508	chr10:10830201-10862214	0	1	0	1	0
509	chr10:11051139-11071801	0	0	0	1	0
510	chr10:15199028-15253891	0	0	1	0	0
511	chr10:17046674-17066136	0	0	0	1	0
512	chr10:17399981-17426802	0	0	0	1	0
513	chr10:17445168-17465603	0	0	0	1	0
514	chr10:19059933-19096725	1	0	0	0	0
515	chr10:19139391-19159541	1	0	0	0	0
516	chr10:19179667-19220027	1	0	0	1	0
517	chr10:19483878-19534648	1	0	0	0	0

518	chr10:20435388-20453974	0	1	0	1	0
519	chr10:21122973-21137085	0	0	0	1	0
520	chr10:21635984-21653215	0	0	0	1	0
521	chr10:25453530-25475116	0	1	0	0	0
522	chr10:25950619-26031202	1	1	1	1	0
523	chr10:26446802-26470529	0	0	0	1	0
524	chr10:28403891-28419153	0	0	0	1	0
525	chr10:28975544-28999462	1	1	0	1	0
526	chr10:29540980-29568081	0	1	0	1	0
527	chr10:29802128-29821750	0	0	0	1	0
528	chr10:30742008-30760215	0	0	0	1	0
529	chr10:33343228-33367822	0	1	0	1	0
530	chr10:33488562-33504404	0	0	0	1	0
531	chr10:34462549-34480912	0	0	0	1	0
532	chr10:34675935-34692300	0	1	0	1	0
533	chr10:35268124-35282378	0	0	0	1	0
534	chr10:36269351-36285655	0	0	0	1	0
535	chr10:37195519-37211331	0	1	0	1	0
536	chr10:37590303-37605598	0	0	0	1	0
537	chr10:39637557-39706499	0	1	0	1	0
538	chr10:40243347-40265718	0	0	0	1	0
539	chr10:40296687-40313110	0	0	0	1	0
540	chr10:40376773-40390922	0	0	0	1	0
541	chr10:41134054-41150213	0	0	0	1	0
542	chr10:43546619-43561133	0	0	0	1	0
543	chr10:45845619-45898441	0	1	1	1	0
544	chr10:46025367-46039601	0	0	0	1	0
545	chr10:46418617-46437091	0	0	0	1	0
546	chr10:50517100-50536671	0	0	0	1	0
547	chr10:50561253-50575523	0	0	0	1	0
548	chr10:50588624-50606037	0	0	0	1	0
549	chr10:51714948-51733505	0	0	0	1	0
550	chr10:53557934-53574181	0	0	0	1	0
551	chr10:53637846-53652226	0	0	0	1	0
552	chr10:53814693-53832051	0	0	0	1	0
553	chr10:53951235-53969660	0	0	0	1	0
554	chr10:55031600-55047873	0	0	0	1	0
555	chr10:55648574-55664878	0	0	0	1	0
556	chr10:56841578-56914302	0	0	1	0	0
557	chr10:61234000-61334000	0	0	0	0	1
558	chr10:63020282-63038882	0	0	0	1	0
559	chr10:63827738-63846276	0	0	0	1	0
560	chr10:64387163-64405477	0	0	0	1	0
561	chr10:64732797-64749013	0	0	0	1	0
562	chr10:67107013-67122141	0	0	0	1	0
563	chr10:67524976-67542293	0	0	0	1	0
564	chr10:69021050-69037280	0	0	0	1	0
565	chr10:69849652-69868047	0	0	0	1	0
566	chr10:70803175-70832339	0	0	0	1	0
567	chr10:70900443-70915623	0	0	0	1	0
568	chr10:70976272-70993645	0	0	0	1	0
569	chr10:71117436-71136791	0	0	0	1	0

570	chr11:3063984-3104918	0	0	0	1	0
571	chr11:12160840-12376453	1	0	0	0	0
572	chr11:12484835-12611952	1	0	0	1	0
573	chr11:13930449-13993752	1	0	0	1	0
574	chr11:14064249-14443644	1	1	1	1	0
575	chr11:16354840-16370952	0	0	0	1	0
576	chr11:19093654-19108952	0	0	0	1	0
577	chr11:22257305-22272695	0	0	0	1	0
578	chr11:24881727-24900350	0	0	0	1	0
579	chr11:25268925-25288307	0	0	0	1	0
580	chr11:26938810-26964723	0	0	0	1	0
581	chr11:27230651-27244977	0	0	0	1	0
582	chr11:33886256-33901654	0	0	0	1	0
583	chr11:36424378-36438457	0	0	0	1	0
584	chr11:39973597-39988785	0	1	0	1	0
585	chr11:43753544-43803409	1	1	0	1	0
586	chr11:45612013-45634591	0	1	0	1	0
587	chr11:47379218-47394414	0	0	0	1	0
588	chr11:53364658-53378820	0	0	0	1	0
589	chr11:55472168-55492340	1	0	0	1	0
590	chr11:55656307-55675960	0	0	0	1	0
591	chr11:57650420-57666833	0	0	0	1	0
592	chr11:57906656-57924061	0	0	0	1	0
593	chr11:62316745-62331873	0	0	0	1	0
594	chr11:62743768-62757813	0	0	0	1	0
595	chr11:63820161-63840631	0	0	0	1	0
596	chr11:63940857-63959398	0	0	0	1	0
597	chr11:66016896-66039478	0	0	0	1	0
598	chr11:67014103-67028377	0	0	0	1	0
599	chr11:67860649-67879920	0	0	0	1	0
600	chr11:68219704-68240329	0	0	0	1	0
601	chr11:69716370-69735927	0	0	0	1	0
602	chr11:70316818-70339414	0	0	0	1	0
603	chr11:70735562-70754292	0	0	0	1	0
604	chr11:70878576-70907366	0	0	0	1	0
605	chr11:71359701-71376900	0	0	0	1	0
606	chr11:71573693-71595373	0	0	0	1	0
607	chr11:71635834-71650918	0	0	0	1	0
608	chr11:71851566-71872089	0	1	0	1	0
609	chr11:72047437-72067182	0	0	0	1	0
610	chr11:72106551-72137605	0	0	0	1	0
611	chr11:72522881-72541357	0	0	0	1	0
612	chr11:73789092-73804297	0	0	0	1	0
613	chr11:74035935-74052104	0	0	0	1	0
614	chr11:74069470-74084693	0	0	0	1	0
615	chr11:75101128-75116406	0	0	0	1	0
616	chr11:75683298-75702882	0	0	0	1	0
617	chr11:75727311-75744728	0	0	0	1	0
618	chr11:76397260-76414607	0	0	0	1	0
619	chr11:76488847-76517182	0	0	0	1	0
620	chr11:76524975-76564395	0	0	0	1	0
621	chr11:76600828-76615150	0	0	0	1	0

622	chr12:2998761-3027097	1	0	0	0	0
623	chr12:3594274-3915665	0	1	0	1	0
624	chr12:4468564-4614485	0	1	0	0	0
625	chr12:4692766-4719305	0	0	0	1	0
626	chr12:4735158-4811076	0	1	0	1	0
627	chr12:4834273-4866587	0	1	0	1	0
628	chr12:5189712-5218750	0	0	0	1	0
629	chr12:5427601-5444889	0	0	0	1	0
630	chr12:6325907-6342036	0	0	0	1	0
631	chr12:6450697-6466008	0	0	0	1	0
632	chr12:6788598-6806943	0	0	0	1	0
633	chr12:6838006-6854251	0	0	0	1	0
634	chr12:8914350-8929731	0	0	0	1	0
635	chr12:9229489-9244700	0	0	0	1	0
636	chr12:10555998-10570064	0	0	0	1	0
637	chr12:11044944-11063199	0	0	0	1	0
638	chr12:12582455-12597916	0	0	0	1	0
639	chr12:13200136-13216606	0	0	0	1	0
640	chr12:13459063-13484640	0	0	0	1	0
641	chr12:15662351-15693720	0	0	0	1	0
642	chr12:16919467-16938946	0	0	0	1	0
643	chr12:17489849-17510344	0	0	0	1	0
644	chr12:17940596-17958843	0	0	0	1	0
645	chr12:18308852-18328618	0	0	0	1	0
646	chr12:20523164-20544952	0	0	0	1	0
647	chr12:20673274-20694736	0	0	0	1	0
648	chr12:22065611-22080901	0	0	0	1	0
649	chr12:23683061-23698250	0	0	0	1	0
650	chr12:24544343-24558507	0	0	0	1	0
651	chr12:27348088-27363442	0	0	0	1	0
652	chr12:29735906-29752167	0	0	0	1	0
653	chr12:33750165-33781129	0	1	0	1	0
654	chr12:34419805-34453853	0	1	0	1	0
655	chr12:35340449-35357768	0	0	0	1	0
656	chr12:37864640-37878604	0	0	0	1	0
657	chr12:38492580-38508717	0	1	0	1	0
658	chr12:41069953-41089411	0	0	0	1	0
659	chr12:47649372-47664633	0	0	0	1	0
660	chr12:49436625-49450759	0	0	0	1	0
661	chr12:50123379-50223379	0	0	0	0	1
662	chr12:57039181-57067335	1	1	0	1	0
663	chr12:57728047-57746353	0	0	0	1	0
664	chr12:59523796-59538847	0	0	0	1	0
665	chr12:60092319-60118042	0	0	0	1	0
666	chr12:62342308-62365953	0	1	0	1	0
667	chr12:65347347-65361702	0	0	0	1	0
668	chr12:65623047-65642397	0	0	0	1	0
669	chr12:65889208-65906716	0	0	0	1	0
670	chr12:66086771-66106496	0	0	0	1	0
671	chr12:66180384-66198764	0	0	0	1	0
672	chr12:71762312-71776576	0	0	0	1	0
673	chr12:72036716-72052024	0	0	0	1	0

674	chr12:73006997-73023143	0	0	0	1	0
675	chr12:73433236-73453552	0	0	0	1	0
676	chr12:73789217-73807793	0	0	0	1	0
677	chr12:74648851-74662973	0	0	0	1	0
678	chr12:74922607-74938779	0	0	0	1	0
679	chr13:5455648-5503485	0	1	0	1	0
680	chr13:8071422-8088672	0	1	0	1	0
681	chr13:8898294-8915552	0	0	0	1	0
682	chr13:12566678-12639288	0	0	1	0	0
683	chr13:14506047-14532119	0	1	0	1	0
684	chr13:14839999-14855302	0	0	0	1	0
685	chr13:15735299-15755603	0	0	0	1	0
686	chr13:16413460-16431921	0	0	0	1	0
687	chr13:19101042-19123224	1	0	0	0	0
688	chr13:19575740-19591292	0	0	0	1	0
689	chr13:20899778-20922374	0	0	0	1	0
690	chr13:26240747-26269093	0	0	0	1	0
691	chr13:27303424-27319653	0	0	0	1	0
692	chr13:31480469-31569361	0	1	0	1	0
693	chr13:32002120-32017530	0	0	0	1	0
694	chr13:34114916-34134555	0	1	0	1	0
695	chr13:36415919-36436405	0	0	0	1	0
696	chr13:41493322-41510685	0	0	0	1	0
697	chr13:42177603-42230449	0	0	0	1	0
698	chr13:42315151-42338869	0	0	0	1	0
699	chr13:42439674-42455900	0	0	0	1	0
700	chr13:42504578-42527292	0	0	0	1	0
701	chr13:42741948-42759134	0	0	0	1	0
702	chr13:42904811-42923169	0	0	0	1	0
703	chr13:43286427-43314595	1	0	0	1	0
704	chr13:43329154-43347615	0	0	0	1	0
705	chr13:43835334-43852766	0	1	0	1	0
706	chr13:44161128-44181587	0	1	0	1	0
707	chr13:44267492-44283777	0	0	0	1	0
708	chr13:46420046-46441532	0	0	0	1	0
709	chr13:46852286-46867429	0	0	0	1	0
710	chr13:48243134-48260594	0	0	0	1	0
711	chr13:48290362-48304649	0	0	0	1	0
712	chr13:48829830-48847318	0	0	0	1	0
713	chr13:49917606-49934974	0	0	0	1	0
714	chr13:51757506-51775552	0	0	0	1	0
715	chr13:51981267-52081267	0	0	0	0	1
716	chr13:56412193-56427554	0	1	0	1	0
717	chr13:61250653-61273311	0	0	0	1	0
718	chr13:61586326-61603713	0	0	0	1	0
719	chr13:61624131-61639463	0	0	0	1	0
720	chr13:61821098-61985322	1	1	0	1	0
721	chr13:62425790-62469200	1	1	0	1	0
722	chr13:62674291-62693866	0	1	0	1	0
723	chr13:62729594-62753659	1	0	0	1	0
724	chr13:63918184-63941647	0	1	0	1	0
725	chr13:64984658-65007227	0	0	0	1	0

726	chr13:65686454-65732925	0	1	0	1	0
727	chr13:65887343-65905405	0	1	0	1	0
728	chr13:66063252-66182471	1	1	0	0	0
729	chr14:3016565-3327175	1	1	0	1	0
730	chr14:3460140-3488090	1	1	0	1	0
731	chr14:3665963-3706365	1	1	0	1	0
732	chr14:5273707-5823919	1	1	1	1	0
733	chr14:8476812-8491867	0	0	0	1	0
734	chr14:8531465-8549051	0	0	0	1	0
735	chr14:8709489-8724657	0	0	0	1	0
736	chr14:10799499-10818873	0	0	0	1	0
737	chr14:14122762-14139226	0	0	0	1	0
738	chr14:14509512-14525773	0	0	0	1	0
739	chr14:14569225-14616196	1	0	0	0	0
740	chr14:15526244-15544727	0	1	0	1	0
741	chr14:16340303-16355666	0	0	0	1	0
742	chr14:17428677-17443984	0	0	0	1	0
743	chr14:20439399-20461882	1	0	0	0	0
744	chr14:20982955-21002416	0	0	0	1	0
745	chr14:22148795-22163977	0	0	0	1	0
746	chr14:25259200-25280525	1	0	0	1	0
747	chr14:27027973-27070609	0	1	0	1	0
748	chr14:30019405-30035608	0	0	0	1	0
749	chr14:34812466-34829674	0	0	0	1	0
750	chr14:38246860-38269642	0	0	0	1	0
751	chr14:42508042-42608042	0	0	0	0	1
752	chr14:42688427-42705654	0	0	0	1	0
753	chr14:42931102-42946298	0	0	0	1	0
754	chr14:45978298-46140677	0	0	1	0	0
755	chr14:46157541-46179001	0	0	0	1	0
756	chr14:46518753-46532998	0	0	0	1	0
757	chr14:47173838-47191312	0	0	0	1	0
758	chr14:47953162-48154923	1	1	0	1	0
759	chr14:49709883-49729376	0	0	0	1	0
760	chr14:50563807-50590240	0	0	0	1	0
761	chr14:52421613-52440045	0	0	0	1	0
762	chr14:53307016-53321352	0	0	0	1	0
763	chr14:54425711-54442184	0	0	0	1	0
764	chr14:54678271-54778271	0	0	0	0	1
765	chr14:55170722-55187160	0	0	0	1	0
766	chr14:55220363-55265128	0	1	0	1	0
767	chr14:55300887-55317061	0	0	0	1	0
768	chr14:57351859-57376567	0	1	0	1	0
769	chr14:58854485-58872669	0	0	0	1	0
770	chr14:59943579-59957687	0	0	0	1	0
771	chr14:60667163-60685655	0	0	0	1	0
772	chr14:60873455-61220668	0	1	1	1	0
773	chr14:61269056-61322155	1	0	0	0	0
774	chr14:62294457-62315626	0	0	0	1	0
775	chr14:62450938-62466187	0	0	0	1	0
776	chr14:62792883-62807257	0	0	0	1	0
777	chr14:63056845-63081444	0	0	0	1	0

778	chr14:63383098-63413032	0	0	0	1	0
779	chr14:63706605-63724252	0	0	0	1	0
780	chr15:3037952-3064622	1	0	0	0	0
781	chr15:3120718-3143191	1	0	0	0	0
782	chr15:3180301-3199098	1	0	0	0	0
783	chr15:3208083-3232669	1	0	0	1	0
784	chr15:5974257-5989500	0	1	0	1	0
785	chr15:6129733-6151188	0	1	0	1	0
786	chr15:7048601-7072026	0	1	0	1	0
787	chr15:7362565-7379088	0	0	0	1	0
788	chr15:7402705-7417142	0	0	0	1	0
789	chr15:10117879-10139795	0	0	0	1	0
790	chr15:12880632-12897101	0	0	0	1	0
791	chr15:13151457-13174206	0	1	0	1	0
792	chr15:15436628-15451960	0	0	0	1	0
793	chr15:15641261-15658720	0	0	0	1	0
794	chr15:16577102-16672475	0	0	1	0	0
795	chr15:17813862-17830431	0	0	0	1	0
796	chr15:18514561-18536131	0	0	0	1	0
797	chr15:20159880-20227982	1	1	0	1	0
798	chr15:27641959-27669745	0	1	0	1	0
799	chr15:29307780-29322941	0	0	0	1	0
800	chr15:31503428-31535844	0	1	1	1	0
801	chr15:31773897-31790275	0	1	0	1	0
802	chr15:32826968-32851381	1	1	0	1	0
803	chr15:33009724-33030312	0	1	0	1	0
804	chr15:33220777-33236117	0	0	0	1	0
805	chr15:33520618-33541295	0	0	0	1	0
806	chr15:34277383-34299055	0	1	0	1	0
807	chr15:37328665-37343910	0	0	0	1	0
808	chr15:41168461-41187249	0	0	0	1	0
809	chr15:41504260-41519503	0	0	0	1	0
810	chr15:42918307-42940137	0	0	0	1	0
811	chr15:43242368-43256590	0	0	0	1	0
812	chr15:44601123-44615203	0	0	0	1	0
813	chr15:45063753-45081310	0	0	0	1	0
814	chr15:45253980-45274419	0	0	0	1	0
815	chr15:45668312-45684849	0	0	0	1	0
816	chr15:45724358-45744902	0	1	0	1	0
817	chr15:51674444-51704309	0	1	0	1	0
818	chr15:54281055-54311140	0	0	0	1	0
819	chr15:55681015-55695137	0	0	0	1	0
820	chr15:56331422-56353036	0	0	0	1	0
821	chr15:59032387-59046578	0	0	0	1	0
822	chr15:59933586-59947764	0	0	0	1	0
823	chr15:60547807-60671102	0	1	0	0	0
824	chr15:61001854-61017347	0	0	0	1	0
825	chr15:61029448-61044689	0	0	0	1	0
826	chr15:61109210-61123464	0	0	0	1	0
827	chr15:61728360-61742688	0	0	0	1	0
828	chr15:62719674-62733702	0	0	0	1	0
829	chr15:64209298-64223430	0	0	0	1	0

830	chr15:64379505-64401082	0	0	0	1	0
831	chr15:64596338-64611660	0	0	0	1	0
832	chr15:64762271-64781707	0	0	0	1	0
833	chr15:65425993-65520363	1	0	0	1	0
834	chr15:65649595-65736184	1	0	0	1	0
835	chr15:65882438-65901803	0	0	0	1	0
836	chr15:67189170-67209912	1	0	0	0	0
837	chr16:3001293-3071927	1	0	0	0	0
838	chr16:3166749-3195481	1	0	0	0	0
839	chr16:4122072-4142266	1	0	0	0	0
840	chr16:4202763-4360649	1	1	0	1	0
841	chr16:4679713-4712774	1	0	0	1	0
842	chr16:4730203-4756572	1	0	0	0	0
843	chr16:9365410-9383909	0	0	0	1	0
844	chr16:10233028-10259656	1	0	0	0	0
845	chr16:13524829-13580705	1	0	0	1	0
846	chr16:14344651-14359809	0	0	0	1	0
847	chr16:15544211-15559695	0	0	0	1	0
848	chr16:16534455-16558791	1	0	0	1	0
849	chr16:16834495-16927041	1	1	0	1	0
850	chr16:18230097-18249599	0	1	0	0	0
851	chr16:18292496-18307521	0	0	0	1	0
852	chr16:21068208-21087779	0	1	0	1	0
853	chr16:22471210-22487608	0	0	0	1	0
854	chr16:25089646-25103974	0	0	0	1	0
855	chr16:25574342-25588457	0	0	0	1	0
856	chr16:28606576-28629470	0	0	0	1	0
857	chr16:28947049-28962349	0	1	0	1	0
858	chr16:30242453-30258800	0	0	0	1	0
859	chr16:33077118-33097576	0	0	0	1	0
860	chr16:38688303-38706705	0	0	0	1	0
861	chr16:40460886-40480391	0	1	0	1	0
862	chr16:41092083-41109690	0	0	0	1	0
863	chr16:41552146-41576790	1	0	0	1	0
864	chr16:42934901-42957231	1	1	0	1	0
865	chr16:43002825-43029093	1	0	0	1	0
866	chr16:43989826-44005997	0	0	0	1	0
867	chr16:44333540-44370693	1	0	0	1	0
868	chr16:44830600-44849076	0	0	0	1	0
869	chr16:47065227-47081547	0	0	0	1	0
870	chr16:50122428-50136522	0	0	0	1	0
871	chr16:50355013-50372387	0	0	0	1	0
872	chr16:50502370-50527100	1	0	0	0	0
873	chr16:51238729-51252910	0	0	0	1	0
874	chr16:53055429-53076002	0	0	0	1	0
875	chr16:53617086-53808291	1	1	0	1	0
876	chr16:53825039-53857099	1	1	0	1	0
877	chr16:53983726-54004312	1	0	0	0	0
878	chr16:54599783-54628045	1	0	0	0	0
879	chr16:54867570-54886077	0	0	0	1	0
880	chr16:55174699-55194080	0	0	0	1	0
881	chr16:55235004-55253122	1	0	0	0	0

882	chr16:56073219-56088264	0	0	0	1	0
883	chr16:56910022-56927337	0	0	0	1	0
884	chr16:56989872-57153933	1	1	0	1	0
885	chr16:57408842-57426588	0	0	0	1	0
886	chr16:57865534-57926159	1	1	0	1	0
887	chr16:58411042-58426287	0	0	0	1	0
888	chr16:58788679-58802788	0	0	0	1	0
889	chr16:58999012-59016171	0	0	0	1	0
890	chr16:59079600-59367624	0	1	1	0	0
891	chr16:59448527-59469072	0	0	0	1	0
892	chr16:59957962-59972217	0	0	0	1	0
893	chr16:60178115-60192315	0	0	0	1	0
894	chr16:60680093-60698616	0	0	0	1	0
895	chr16:60763394-60782896	0	0	0	1	0
896	chr16:61435950-61459453	0	0	0	1	0
897	chr16:61747545-61763607	0	0	0	1	0
898	chr16:61888977-62570175	1	1	1	1	0
899	chr17:2999333-3019356	0	0	0	1	0
900	chr17:7508321-7522486	0	0	0	1	0
901	chr17:7543410-7557469	0	0	0	1	0
902	chr17:12036771-12053066	0	0	0	1	0
903	chr17:14375148-14390373	0	0	0	1	0
904	chr17:14478371-14524100	0	0	0	1	0
905	chr17:14721081-14740565	0	0	0	1	0
906	chr17:16633701-16648142	0	0	0	1	0
907	chr17:16843338-16858484	0	0	0	1	0
908	chr17:17783579-17800802	0	0	0	1	0
909	chr17:19195500-19295500	0	0	0	0	1
910	chr17:24430019-24801805	1	1	0	1	0
911	chr17:27552902-27570077	0	0	0	1	0
912	chr17:28959798-28979156	0	0	0	1	0
913	chr17:29299966-29315191	0	0	0	1	0
914	chr17:37430891-37459953	0	1	0	1	0
915	chr17:40308061-40325334	0	0	0	1	0
916	chr17:40669512-40695807	0	1	0	1	0
917	chr17:40738609-40803594	1	1	0	1	0
918	chr17:42212038-42226244	0	0	0	1	0
919	chr17:42383490-42430052	1	1	0	1	0
920	chr17:42657160-42679688	0	0	0	1	0
921	chr17:43143848-43158953	0	0	0	1	0
922	chr17:43354741-43370032	0	0	0	1	0
923	chr17:43407030-43424401	0	0	0	1	0
924	chr17:44649571-47075941	0	1	1	1	0
925	chr17:48341612-48360038	0	1	0	1	0
926	chr17:55557152-55576708	0	1	0	1	0
927	chr17:56242957-56260422	0	0	0	1	0
928	chr17:57528463-57552116	0	0	0	1	0
929	chr17:57565626-57583155	0	0	0	1	0
930	chr17:59701615-59953467	1	1	1	1	0
931	chr17:60450932-60491108	1	1	0	1	0
932	chr17:60497325-60529106	1	0	0	0	0
933	chr17:60549745-60576685	1	0	0	1	0

934	chr17:61959285-61977518	0	0	0	1	0
935	chr17:63112279-63127508	0	0	0	1	0
936	chr17:63762527-63776654	0	0	0	1	0
937	chr17:64662082-64711046	1	1	0	1	0
938	chr17:64871855-64888360	0	0	0	1	0
939	chr17:65109201-65130654	0	0	0	1	0
940	chr17:66305148-66327424	0	1	0	1	0
941	chr17:66488762-66504892	0	0	0	1	0
942	chr18:5569059-5592288	1	0	0	1	0
943	chr18:7724276-7788068	0	1	1	1	0
944	chr18:7926681-7946020	0	0	0	1	0
945	chr18:10528135-10542456	0	0	0	1	0
946	chr18:14022070-14040314	0	0	0	1	0
947	chr18:14241032-14265858	1	0	0	0	0
948	chr18:14298066-14435292	1	1	1	1	0
949	chr18:14481178-14510793	1	0	0	0	0
950	chr18:16703375-16718619	0	0	0	1	0
951	chr18:21338918-21835839	1	1	1	1	0
952	chr18:22159071-22189060	1	0	0	0	0
953	chr18:23355987-23370142	0	0	0	1	0
954	chr18:27162829-27182129	0	0	0	1	0
955	chr18:27884659-27903558	0	0	0	1	0
956	chr18:28060794-28081053	0	0	0	1	0
957	chr18:28301691-28324899	1	0	0	0	0
958	chr18:28442219-28462365	1	0	0	0	0
959	chr18:30261998-30282714	0	0	0	1	0
960	chr18:31181738-31195964	0	0	0	1	0
961	chr18:31476527-31493717	0	1	0	1	0
962	chr18:31996550-32010783	0	0	0	1	0
963	chr18:33245026-33285097	1	0	0	0	0
964	chr18:34889733-34904805	0	0	0	1	0
965	chr18:35033590-35047709	0	0	0	1	0
966	chr18:36025667-36042040	0	0	0	1	0
967	chr18:40405081-40490177	1	0	0	1	0
968	chr18:41730760-41770613	0	1	0	0	0
969	chr18:41942338-41958593	0	0	0	1	0
970	chr18:42333258-42397202	1	0	0	0	0
971	chr18:42635684-42671820	0	1	0	1	0
972	chr18:43137502-43154921	0	0	0	1	0
973	chr18:43326509-43529058	1	1	0	1	0
974	chr18:43645438-43661468	0	1	0	1	0
975	chr18:43720707-43997014	1	1	0	1	0
976	chr18:44198785-44403557	1	1	0	1	0
977	chr18:45088326-45105806	0	0	0	1	0
978	chr18:47353084-47368492	0	0	0	1	0
979	chr18:47825751-47841046	0	0	0	1	0
980	chr18:48551883-48573253	0	0	0	1	0
981	chr18:49150749-49165028	0	0	0	1	0
982	chr18:49323954-49339109	0	0	0	1	0
983	chr18:50839157-50855488	0	0	0	1	0
984	chr18:50919047-50936291	0	0	0	1	0
985	chr18:50961954-50977153	0	0	0	1	0

986	chr18:52096032-52181816	1	1	0	1	0
987	chr18:52852471-52869671	0	0	0	1	0
988	chr18:52915458-52930555	0	1	0	1	0
989	chr18:53186160-53205517	0	0	0	1	0
990	chr18:54236934-54257648	0	0	0	1	0
991	chr18:54363670-54378968	0	0	0	1	0
992	chr18:54483634-54502051	0	0	0	1	0
993	chr18:54568631-54713153	0	1	0	0	0
994	chr18:56851100-56870510	0	0	0	1	0
995	chr18:56904881-56922003	0	0	0	1	0
996	chr18:57091278-57106857	0	0	0	1	0
997	chr18:57291663-57437148	1	1	0	1	0
998	chr18:57529789-57547000	0	0	0	1	0
999	chr18:57686098-57715650	1	1	0	1	0
1000	chr18:58778305-58872314	1	0	0	1	0
1001	chr19:4713580-4753215	0	1	0	1	0
1002	chr19:7133652-7150126	0	0	0	1	0
1003	chr19:7460153-7478628	0	0	0	1	0
1004	chr19:7815104-7834508	0	0	0	1	0
1005	chr19:8981201-9000530	0	0	0	1	0
1006	chr19:9706001-9728916	1	0	0	0	0
1007	chr19:10186912-10207641	0	0	0	1	0
1008	chr19:10899196-10914306	0	0	0	1	0
1009	chr19:22212031-22235939	1	0	0	1	0
1010	chr19:22303497-22329887	1	0	0	0	0
1011	chr19:22400293-22510098	1	1	0	1	0
1012	chr19:22728152-22762251	0	1	0	1	0
1013	chr19:22835008-23349051	1	1	1	1	0
1014	chr19:23452477-23468869	0	0	0	1	0
1015	chr19:24154238-24175158	0	1	0	1	0
1016	chr19:24438882-24482427	1	0	0	1	0
1017	chr19:27150906-27171316	0	0	0	1	0
1018	chr19:28273253-28289556	0	0	0	1	0
1019	chr19:31124513-31141923	0	0	0	1	0
1020	chr19:31295964-31312370	0	0	0	1	0
1021	chr19:32521924-32541328	0	0	0	1	0
1022	chr19:33298401-33312662	0	0	0	1	0
1023	chr19:35000393-35018773	0	0	0	1	0
1024	chr19:35233765-35251120	0	0	0	1	0
1025	chr19:35672172-35686226	0	0	0	1	0
1026	chr19:35693084-35709464	0	0	0	1	0
1027	chr19:37387758-37407111	0	0	0	1	0
1028	chr19:37974218-37988400	0	0	0	1	0
1029	chr19:38504441-38521781	0	0	0	1	0
1030	chr19:39025819-39050555	0	1	0	1	0
1031	chr19:40330761-40353262	0	0	0	1	0
1032	chr19:41521243-41547051	0	0	0	1	0
1033	chr19:46029656-46045865	0	0	0	1	0
1034	chr19:48211336-48229750	0	0	0	1	0
1035	chr19:48951209-48968622	0	0	0	1	0
1036	chr19:50427780-50445084	0	0	0	1	0
1037	chr19:51555456-51601370	0	0	1	0	0

1038	chr19:53617193-53634673	0	0	0	1	0
1039	chr19:53680186-53696466	0	0	0	1	0
1040	chr19:53733480-53757215	0	0	0	1	0
1041	chr19:53778703-53795012	0	1	0	1	0
1042	chr19:53806220-53824071	0	0	0	1	0
1043	chr19:53865579-53880780	0	0	0	1	0
1044	chr19:54067099-54084536	0	0	0	1	0
1045	chr19:54817785-54831995	0	0	0	1	0
1046	chr19:54921621-54936843	0	0	0	1	0
1047	chr19:55578395-55595716	0	0	0	1	0
1048	chr19:55823999-55842317	0	0	0	1	0
1049	chr19:55895167-55929662	1	0	0	0	0
1050	chr19:56098045-56116444	0	0	0	1	0
1051	chr19:56565459-56586816	0	0	0	1	0
1052	chr19:56655669-56669803	0	0	0	1	0
1053	chr20:7636174-7734734	0	1	0	1	0
1054	chr20:8025788-8043079	0	0	0	1	0
1055	chr20:10480479-10495032	0	0	0	1	0
1056	chr20:10665260-10680404	0	0	0	1	0
1057	chr20:10856779-10877319	0	0	0	1	0
1058	chr20:12623257-12643774	0	0	0	1	0
1059	chr20:12978847-12998254	0	0	0	1	0
1060	chr20:13881129-13895547	0	0	0	1	0
1061	chr20:15145054-15245054	0	0	0	0	1
1062	chr20:19142081-19415399	0	1	0	0	0
1063	chr20:21534740-21552134	0	0	0	1	0
1064	chr20:24938584-24955913	0	0	0	1	0
1065	chr20:25361623-25377935	0	0	0	1	0
1066	chr20:26373123-26396803	0	1	0	1	0
1067	chr20:28572840-28595522	0	0	0	1	0
1068	chr20:30103820-30119155	0	0	0	1	0
1069	chr20:31471500-31488675	0	0	0	1	0
1070	chr20:31846506-31865769	0	0	0	1	0
1071	chr20:34027842-34041988	0	0	0	1	0
1072	chr20:34841343-34855651	0	0	0	1	0
1073	chr20:35028805-35047379	0	0	0	1	0
1074	chr20:49733126-49753474	0	0	0	1	0
1075	chr20:50552585-50585912	0	1	0	1	0
1076	chr20:50667010-50702604	0	1	0	1	0
1077	chr20:50741925-50758278	0	1	0	1	0
1078	chr20:52970170-52986445	0	0	0	1	0
1079	chr20:53979570-54001242	0	0	0	1	0
1080	chr20:54783492-54800817	0	0	0	1	0
1081	chr20:56250024-56304307	1	1	0	1	0
1082	chr20:56657784-56677187	0	0	0	1	0
1083	chr20:57143407-57185508	1	1	0	1	0
1084	chr20:58650237-58665445	0	0	0	1	0
1085	chr20:59396603-59457292	0	1	0	1	0
1086	chr20:60943156-60963777	0	0	0	1	0
1087	chr21:2997898-3034595	1	0	0	0	0
1088	chr21:4584254-4598630	0	0	0	1	0
1089	chr21:6222011-6243199	0	0	0	1	0

1090	chr21:7029268-7043342	0	0	0	1	0
1091	chr21:7108668-7127942	0	1	0	1	0
1092	chr21:7720608-7734833	0	0	0	1	0
1093	chr21:9438728-9455909	0	0	0	1	0
1094	chr21:10398719-10422677	0	0	0	1	0
1095	chr21:10956482-10974873	0	0	0	1	0
1096	chr21:12909313-12925563	0	0	0	1	0
1097	chr21:16322057-16339510	0	0	0	1	0
1098	chr21:21444975-21465359	0	0	0	1	0
1099	chr21:22110514-22125766	0	0	0	1	0
1100	chr21:22470423-22494301	0	0	0	1	0
1101	chr21:23319121-23336553	0	0	0	1	0
1102	chr21:25802859-25819159	0	0	0	1	0
1103	chr21:29706307-29779822	0	1	1	1	0
1104	chr21:30127964-30150147	0	1	0	1	0
1105	chr21:30160320-30180764	1	0	0	0	0
1106	chr21:30186025-30323483	1	0	0	1	0
1107	chr21:30337946-30358280	1	0	0	0	0
1108	chr21:30818420-30838844	0	1	0	1	0
1109	chr21:31066711-31162768	1	1	0	1	0
1110	chr21:31234283-31288324	1	0	0	0	0
1111	chr21:31548110-31613003	1	1	0	1	0
1112	chr21:31715773-31733999	1	0	0	0	0
1113	chr21:31944078-31982244	1	0	0	0	0
1114	chr21:32294229-32412270	1	1	0	1	0
1115	chr21:33267908-33300866	1	1	0	1	0
1116	chr21:33560201-33597890	0	1	0	1	0
1117	chr21:33760141-33848940	0	1	0	1	0
1118	chr21:34643897-34671641	0	0	0	1	0
1119	chr21:35461060-35479646	0	0	0	1	0
1120	chr21:35760915-35777135	0	0	0	1	0
1121	chr21:39022186-39036340	0	0	0	1	0
1122	chr21:40295437-40315272	0	0	0	1	0
1123	chr21:40749346-40774394	1	1	0	1	0
1124	chr21:43520019-43539398	0	0	0	1	0
1125	chr21:43816021-43843878	1	0	0	1	0
1126	chr21:43850242-43903051	1	1	0	1	0
1127	chr21:44044402-44090600	0	0	1	0	0
1128	chr21:44853310-44867370	0	0	0	1	0
1129	chr21:45127434-45148875	0	1	0	1	0
1130	chr21:46111667-46129204	0	0	0	1	0
1131	chr21:47949855-47964242	0	0	0	1	0
1132	chr21:48194894-49784305	0	1	0	0	0
1133	chr21:49793030-49827716	1	0	0	1	0
1134	chr21:50129928-50145149	0	1	0	1	0
1135	chr21:51221546-51246107	0	1	0	1	0
1136	chr21:52168952-52184155	0	0	0	1	0
1137	chr21:52282027-52297127	0	0	0	1	0
1138	chr21:53567855-53582047	0	0	0	1	0
1139	chr22:3158055-3175545	0	0	0	1	0
1140	chr22:3365755-3380074	0	0	0	1	0
1141	chr22:4707704-4731201	0	1	0	1	0

1142	chr22:5347342-5363492	0	0	0	1	0
1143	chr22:6408536-6422608	0	0	0	1	0
1144	chr22:12504654-12522292	0	0	0	1	0
1145	chr22:17168881-17186351	0	0	0	1	0
1146	chr22:17830166-17857049	0	1	0	1	0
1147	chr22:18060990-18076998	0	0	0	1	0
1148	chr22:18467887-18486090	0	0	0	1	0
1149	chr22:21475119-21501904	0	1	0	1	0
1150	chr22:23414672-23432957	0	1	0	1	0
1151	chr22:32683500-32783500	0	0	0	0	1
1152	chr22:34295553-34309586	0	0	0	1	0
1153	chr22:38399244-38425067	0	0	0	1	0
1154	chr22:38705399-38733271	0	1	0	1	0
1155	chr22:38764222-38811016	0	1	0	1	0
1156	chr22:40345255-40359603	0	0	0	1	0
1157	chr22:40514699-40529823	0	0	0	1	0
1158	chr22:40554445-40582326	0	1	0	1	0
1159	chr22:42396050-42411223	0	1	0	1	0
1160	chr22:43323523-43341869	0	0	0	1	0
1161	chr22:46895311-46926282	0	1	0	1	0
1162	chr22:48915507-48932901	0	0	0	1	0
1163	chr22:53883081-53899528	0	0	0	1	0
1164	chr22:54237126-54272402	0	1	0	1	0
1165	chr22:54484650-54500831	0	0	0	1	0
1166	chr22:56690076-56739245	1	0	0	0	0
1167	chr22:57878034-58017926	0	0	1	0	0
1168	chr22:58071103-58094719	0	0	0	1	0
1169	chr22:63223261-63237605	0	0	0	1	0
1170	chr22:63906652-63921982	0	0	0	1	0
1171	chr22:63934987-63950303	0	0	0	1	0
1172	chr22:64375529-64394499	1	0	0	0	0
1173	chr23:4293251-4316755	0	0	0	1	0
1174	chr23:5645028-5660328	0	1	0	1	0
1175	chr23:6160278-6175714	0	0	0	1	0
1176	chr23:8436970-8452247	0	0	0	1	0
1177	chr23:9941671-9960191	0	0	0	1	0
1178	chr23:12966555-12985087	0	0	0	1	0
1179	chr23:13662209-13679673	0	0	0	1	0
1180	chr23:14435704-14453159	0	0	0	1	0
1181	chr23:17224457-17242669	0	0	0	1	0
1182	chr23:18164633-18216806	1	1	0	1	0
1183	chr23:20793152-20808341	0	0	0	1	0
1184	chr23:21598691-21616955	0	0	0	1	0
1185	chr23:21709002-21726652	0	0	0	1	0
1186	chr23:22518403-22545279	0	0	0	1	0
1187	chr23:23532155-23793196	0	1	1	1	0
1188	chr23:25229279-25243449	0	0	0	1	0
1189	chr23:26795168-26809601	0	0	0	1	0
1190	chr23:31538871-31563401	0	0	0	1	0
1191	chr23:31915013-31929176	0	0	0	1	0
1192	chr23:34830389-34852822	0	0	0	1	0
1193	chr23:35640565-35656993	0	0	0	1	0

1194	chr23:36475109-36500940	0	1	0	1	0
1195	chr23:37338766-37358105	1	0	0	1	0
1196	chr23:38163807-38181305	0	0	0	1	0
1197	chr23:38256705-38270710	0	0	0	1	0
1198	chr23:43516328-43566328	0	0	0	0	1
1199	chr23:44755150-44772431	0	0	0	1	0
1200	chr23:45552460-45571863	0	0	0	1	0
1201	chr23:46339392-46353492	0	0	0	1	0
1202	chr23:48531185-48547650	0	0	0	1	0
1203	chr23:48746193-48765531	0	0	0	1	0
1204	chr23:48927046-48944406	0	0	0	1	0
1205	chr23:50606148-50625430	0	1	0	1	0
1206	chr23:51242758-51265657	0	1	0	1	0
1207	chr23:52028057-52045391	0	0	0	1	0
1208	chr23:52717248-52732392	0	0	0	1	0
1209	chr23:54473811-54487874	0	0	0	1	0
1210	chr23:55211210-55225447	0	0	0	1	0
1211	chr24:2996041-3022534	1	0	0	0	0
1212	chr24:7580001-7638310	0	1	0	1	0
1213	chr24:10331497-10352029	0	0	0	1	0
1214	chr24:11287506-11305807	0	1	0	1	0
1215	chr24:13217761-13244178	1	0	0	0	0
1216	chr24:13728383-13746919	0	0	0	1	0
1217	chr24:17409459-17425978	0	0	0	1	0
1218	chr24:17612090-17633154	0	0	0	1	0
1219	chr24:17649770-17699770	0	0	0	0	1
1220	chr24:20658974-20677377	0	0	0	1	0
1221	chr24:22224078-22384923	1	1	0	1	0
1222	chr24:25251438-25265553	0	0	0	1	0
1223	chr24:29006520-29024775	0	0	0	1	0
1224	chr24:30496879-30515244	0	0	0	1	0
1225	chr24:33557098-33572403	0	0	0	1	0
1226	chr24:33944587-33959893	0	0	0	1	0
1227	chr24:34342251-34360836	0	0	0	1	0
1228	chr24:34720415-34734644	0	0	0	1	0
1229	chr24:34763106-34778158	0	0	0	1	0
1230	chr24:37068139-37084437	0	0	0	1	0
1231	chr24:37807301-37841454	0	1	0	1	0
1232	chr24:37917004-37932279	0	0	0	1	0
1233	chr24:38040350-38058560	0	0	0	1	0
1234	chr24:38363747-38387346	0	1	0	1	0
1235	chr24:38961222-38975420	0	0	0	1	0
1236	chr24:40518797-40540204	0	0	0	1	0
1237	chr24:40706172-40726599	0	0	0	1	0
1238	chr24:43604641-43618889	0	0	0	1	0
1239	chr24:48024558-48046099	0	1	0	1	0
1240	chr24:48118012-48145840	1	0	0	0	0
1241	chr24:48419924-48435607	0	0	0	1	0
1242	chr24:48839419-48857654	0	0	0	1	0
1243	chr24:48980455-49019805	0	1	0	1	0
1244	chr24:49777249-49794796	0	0	0	1	0
1245	chr25:2996041-3051488	1	0	0	0	0

1246	chr25:5203966-5223488	0	1	0	1	0
1247	chr25:8520832-8620832	0	0	0	0	1
1248	chr25:13894361-13911774	0	0	0	1	0
1249	chr25:14001997-14020060	0	0	0	1	0
1250	chr25:15160805-15178293	0	0	0	1	0
1251	chr25:17226533-17248954	0	0	0	1	0
1252	chr25:17734127-17751266	0	0	0	1	0
1253	chr25:17938108-17954435	0	0	0	1	0
1254	chr25:18511545-18527999	0	0	0	1	0
1255	chr25:18547584-18562736	0	0	0	1	0
1256	chr25:21510856-21525024	0	0	0	1	0
1257	chr25:22022943-22052833	0	1	0	0	0
1258	chr25:22316792-22339391	0	0	0	1	0
1259	chr25:22697599-22747936	1	0	0	0	0
1260	chr25:22800112-22873921	1	0	0	0	0
1261	chr25:24270181-24285516	0	0	0	1	0
1262	chr25:26493083-26508484	0	0	0	1	0
1263	chr25:29028107-29046336	0	0	0	1	0
1264	chr25:29092563-29109829	0	0	0	1	0
1265	chr25:29375057-29389299	0	0	0	1	0
1266	chr25:30776515-30790730	0	0	0	1	0
1267	chr25:33718933-33734618	0	0	0	1	0
1268	chr25:35940265-35959709	0	0	0	1	0
1269	chr25:36755890-36775308	0	0	0	1	0
1270	chr25:37299780-37314967	0	0	0	1	0
1271	chr25:37504601-37523004	0	0	0	1	0
1272	chr25:38818061-38835462	0	1	0	1	0
1273	chr25:40120259-40140724	0	0	0	1	0
1274	chr25:40532110-40551881	0	0	0	1	0
1275	chr25:40936619-40953876	0	0	0	1	0
1276	chr25:41158253-41178893	0	0	0	1	0
1277	chr25:42915653-42951864	0	0	0	1	0
1278	chr25:48051062-48118154	1	1	0	1	0
1279	chr25:48773165-48791431	0	0	0	1	0
1280	chr25:49291603-49312459	0	0	0	1	0
1281	chr25:49370551-49384892	0	0	0	1	0
1282	chr25:49643673-49657950	0	0	0	1	0
1283	chr25:50600629-50616865	0	0	0	1	0
1284	chr25:52332995-52347153	0	0	0	1	0
1285	chr25:52372248-52388348	0	0	0	1	0
1286	chr25:53000035-53020550	1	1	0	1	0
1287	chr25:53114029-53201829	1	1	0	1	0
1288	chr25:53216808-53251504	0	1	0	1	0
1289	chr25:53261470-53293387	1	0	0	0	0
1290	chr25:54173461-54189557	0	0	0	1	0
1291	chr26:4244419-4260637	0	0	0	1	0
1292	chr26:4750223-4765436	0	0	0	1	0
1293	chr26:5705921-5727204	0	1	0	0	0
1294	chr26:6246198-6261328	0	0	0	1	0
1295	chr26:11270735-11287999	0	0	0	1	0
1296	chr26:12440280-12479237	1	0	0	0	0
1297	chr26:15764819-15784418	0	1	0	1	0

1298	chr26:19354650-19368670	0	0	0	1	0
1299	chr26:20022823-20078322	1	0	0	1	0
1300	chr26:21951766-22000083	1	1	0	1	0
1301	chr26:22257442-22271591	0	0	0	1	0
1302	chr26:23258102-23273488	0	0	0	1	0
1303	chr26:24017860-24031965	0	0	0	1	0
1304	chr26:24108238-24130705	0	0	0	1	0
1305	chr26:25392820-25406925	0	0	0	1	0
1306	chr26:28042205-29096517	1	1	0	1	0
1307	chr26:29142411-29621741	1	1	0	1	0
1308	chr26:29628536-29731810	1	0	0	1	0
1309	chr26:29780834-29830103	1	1	0	1	0
1310	chr26:29840689-29859218	1	0	0	0	0
1311	chr26:29890733-29919689	1	0	0	0	0
1312	chr26:30007328-30049569	0	1	0	1	0
1313	chr26:30223896-30297483	0	1	1	1	0
1314	chr26:30354842-30632001	1	1	0	1	0
1315	chr26:30671363-30697966	1	1	0	1	0
1316	chr26:31890098-32000206	1	1	0	1	0
1317	chr26:34221628-34697632	1	1	0	1	0
1318	chr26:35166197-35183496	0	0	0	1	0
1319	chr26:35485091-35503359	0	1	0	1	0
1320	chr26:36197259-36219650	0	0	0	1	0
1321	chr26:37355730-37382534	0	0	0	1	0
1322	chr26:37388335-37405690	0	1	0	1	0
1323	chr26:39321021-39337404	0	0	0	1	0
1324	chr26:41456636-41471702	0	0	0	1	0
1325	chr26:41783265-41803890	0	0	0	1	0
1326	chr27:3516987-3534477	0	0	0	1	0
1327	chr27:3713564-3729764	0	0	0	1	0
1328	chr27:5352510-5368763	0	0	0	1	0
1329	chr27:5688193-5709849	0	0	0	1	0
1330	chr27:6193651-6208753	0	0	0	1	0
1331	chr27:6523059-6587460	0	1	0	1	0
1332	chr27:8934072-8963399	1	0	0	0	0
1333	chr27:8988491-9008754	1	0	0	0	0
1334	chr27:9015577-9076710	1	1	0	0	0
1335	chr27:9356562-9381857	0	0	0	1	0
1336	chr27:10013694-10031188	0	0	0	1	0
1337	chr27:12194655-12209009	0	0	0	1	0
1338	chr27:13728642-13742755	0	0	0	1	0
1339	chr27:16136865-16217123	1	1	0	1	0
1340	chr27:17460450-17502000	0	0	1	0	0
1341	chr27:18253370-18271051	0	0	0	1	0
1342	chr27:20927218-20955534	0	1	0	1	0
1343	chr27:21527548-21541669	0	0	0	1	0
1344	chr27:22225560-22239611	0	0	0	1	0
1345	chr27:22278103-22293507	0	0	0	1	0
1346	chr27:24368392-24384657	0	0	0	1	0
1347	chr27:24467197-24494365	0	0	0	1	0
1348	chr27:28693064-29201491	1	1	1	1	0
1349	chr27:31079289-31094552	0	1	0	1	0

1350	chr27:32646539-32661837	0	0	0	1	0
1351	chr27:32866210-32884800	0	0	0	1	0
1352	chr27:33181401-33200886	0	0	0	1	0
1353	chr27:35040923-35056086	0	0	0	1	0
1354	chr27:36120045-36136371	0	0	0	1	0
1355	chr27:36554185-36570318	0	0	0	1	0
1356	chr27:37699945-37716184	0	0	0	1	0
1357	chr27:38012742-38035493	0	1	0	1	0
1358	chr27:38321522-38414452	1	1	0	1	0
1359	chr27:38433606-38480859	1	0	0	1	0
1360	chr27:38548124-38569634	0	1	0	1	0
1361	chr27:38679579-38700107	0	1	0	1	0
1362	chr27:38735159-38760791	0	1	0	1	0
1363	chr27:40735022-40757615	1	0	0	0	0
1364	chr27:41357369-41375797	0	0	0	1	0
1365	chr27:41673582-41694046	0	0	0	1	0
1366	chr27:41779068-41798631	0	0	0	1	0
1367	chr27:43149899-43173454	0	0	0	1	0
1368	chr28:4149457-4163636	0	0	0	1	0
1369	chr28:6072061-6088379	0	0	0	1	0
1370	chr28:6229232-6255235	0	0	0	1	0
1371	chr28:10537470-10554728	0	1	0	1	0
1372	chr28:12551993-12567302	0	0	0	1	0
1373	chr28:16079113-16114951	1	1	0	1	0
1374	chr28:16855320-16874758	0	0	0	1	0
1375	chr28:18683324-18698806	0	1	0	1	0
1376	chr28:27344074-27444074	0	0	0	0	1
1377	chr28:27944376-27975599	0	1	0	1	0
1378	chr28:29898178-29914541	0	0	0	1	0
1379	chr28:31219892-31236163	0	0	0	1	0
1380	chr28:31475199-31489476	0	1	0	1	0
1381	chr28:35163298-35182806	0	0	0	1	0
1382	chr28:35216839-35273034	1	1	0	1	0
1383	chr28:35278039-35297247	1	0	0	0	0
1384	chr28:36429379-36445175	0	0	0	1	0
1385	chr28:38017124-38033327	0	0	0	1	0
1386	chr28:40307797-40322048	0	0	0	1	0
1387	chr28:40581808-40599287	0	0	0	1	0
1388	chr28:42264007-42281489	0	0	0	1	0
1389	chr28:42636997-42681226	0	0	0	1	0
1390	chr28:42836680-42851920	0	0	0	1	0
1391	chr28:43666254-43680763	0	0	0	1	0
1392	chr29:4580672-4627657	0	1	0	1	0
1393	chr29:4876405-4897046	0	1	0	1	0
1394	chr29:5149865-5166250	0	0	0	1	0
1395	chr29:5229580-5246712	0	0	0	1	0
1396	chr29:6016326-6040991	0	1	0	1	0
1397	chr29:6087214-6108044	0	0	0	1	0
1398	chr29:9026577-9047041	0	1	0	1	0
1399	chr29:10178566-10196957	0	0	0	1	0
1400	chr29:10868501-10886665	0	1	0	1	0
1401	chr29:14852409-14868955	0	0	0	1	0

1402	chr29:15723970-15739103	0	1	0	1	0
1403	chr29:16132607-16152968	0	0	0	1	0
1404	chr29:16785012-16799252	0	0	0	1	0
1405	chr29:21688863-21705008	0	0	0	1	0
1406	chr29:22218301-22245066	0	1	0	1	0
1407	chr29:23766218-23781306	0	0	0	1	0
1408	chr29:25321856-25336111	0	0	0	1	0
1409	chr29:26323879-26423879	0	0	0	0	1
1410	chr29:26627316-26641302	0	0	0	1	0
1411	chr29:26668462-26686828	0	0	0	1	0
1412	chr29:27654616-27675067	0	0	0	1	0
1413	chr29:28004162-28020586	0	0	0	1	0
1414	chr29:28845005-28859207	0	0	0	1	0
1415	chr29:29546185-29560253	0	0	0	1	0
1416	chr29:30171837-30191244	0	0	0	1	0
1417	chr29:30223215-30250697	0	1	0	1	0
1418	chr29:38332626-38349202	0	0	0	1	0
1419	chr29:38369226-38410415	0	0	1	0	0
1420	chr29:38476532-38496980	0	0	0	1	0
1421	chr29:38818009-38871017	0	1	0	0	0
1422	chr29:39292863-39307088	0	0	0	1	0
1423	chr29:43034924-43051255	0	0	0	1	0
1424	chr29:43522354-43536600	0	0	0	1	0
1425	chr29:44400664-44418161	0	0	0	1	0
1426	chr29:44763817-44827901	1	0	0	0	0
1427	chr30:3157321-3175915	0	0	0	1	0
1428	chr30:8843151-8862805	0	0	0	1	0
1429	chr30:14783654-14801972	0	0	0	1	0
1430	chr30:15531426-15548738	0	0	0	1	0
1431	chr30:20095660-20115028	0	0	0	1	0
1432	chr30:25187620-25206034	0	0	0	1	0
1433	chr30:26026052-26041145	0	0	0	1	0
1434	chr30:29040635-29057910	0	1	0	1	0
1435	chr30:29434282-29448467	0	0	0	1	0
1436	chr30:30946870-30961059	0	0	0	1	0
1437	chr30:33403247-33424718	0	0	0	1	0
1438	chr30:33922447-33938540	0	0	0	1	0
1439	chr30:34967859-34983119	0	0	0	1	0
1440	chr30:35759905-35782123	0	0	0	1	0
1441	chr30:39078045-39092112	0	0	0	1	0
1442	chr30:40464105-40492810	1	0	0	0	0
1443	chr30:40716071-40730342	0	0	0	1	0
1444	chr30:40782873-40833444	1	1	0	1	0
1445	chr30:42153993-42168116	0	0	0	1	0
1446	chr31:6271310-6292704	0	1	0	1	0
1447	chr31:12953492-13053492	0	0	0	0	1
1448	chr31:13346932-13365235	0	0	0	1	0
1449	chr31:17012486-17040714	0	0	0	1	0
1450	chr31:20134172-20152685	0	0	0	1	0
1451	chr31:21597044-21612400	0	0	0	1	0
1452	chr31:24148062-24162183	0	0	0	1	0
1453	chr31:25440665-25468663	0	1	0	1	0

1454	chr31:26577955-26596665	0	0	0	1	0
1455	chr31:30136024-30150016	0	0	0	1	0
1456	chr31:30221295-30242782	0	0	0	1	0
1457	chr31:31845799-31859895	0	0	0	1	0
1458	chr31:33431847-33486855	1	1	0	1	0
1459	chr31:34532053-34562863	0	0	0	1	0
1460	chr31:36392100-36410582	0	0	0	1	0
1461	chr31:38195449-38543159	0	1	1	1	0
1462	chr31:38943109-38969932	0	0	0	1	0
1463	chr31:39167215-39186718	0	0	0	1	0
1464	chr31:39268869-39288525	0	0	0	1	0
1465	chr31:39583913-39618833	0	0	0	1	0
1466	chr31:39861272-39877517	0	0	0	1	0
1467	chr31:40458693-40473730	0	1	0	1	0
1468	chr31:40617054-40663817	1	1	0	1	0
1469	chr31:41401230-41415344	0	0	0	1	0
1470	chr32:4541887-4557149	0	0	0	1	0
1471	chr32:5281053-5297384	0	0	0	1	0
1472	chr32:5625474-5639632	0	0	0	1	0
1473	chr32:5899651-5913703	0	0	0	1	0
1474	chr32:7004303-7019780	0	0	0	1	0
1475	chr32:7245599-7268119	0	0	0	1	0
1476	chr32:7834098-7851652	0	0	0	1	0
1477	chr32:8079713-8094030	0	0	0	1	0
1478	chr32:8395450-8419186	0	0	0	1	0
1479	chr32:10790053-10805530	0	0	0	1	0
1480	chr32:11761431-11778884	0	0	0	1	0
1481	chr32:17786069-17944153	0	0	1	0	0
1482	chr32:21857739-21871995	0	0	0	1	0
1483	chr32:22730937-22747320	0	1	0	1	0
1484	chr32:25482769-25496907	0	0	0	1	0
1485	chr32:28482851-28501002	0	0	0	1	0
1486	chr32:35230530-35249889	0	0	0	1	0
1487	chr32:41384081-41464581	1	0	0	1	0
1488	chr32:41592939-41721789	1	0	0	1	0
1489	chr33:3078240-3097653	0	0	0	1	0
1490	chr33:5009487-5023639	0	0	0	1	0
1491	chr33:5280295-5301393	1	0	0	0	0
1492	chr33:5355779-5370994	0	0	0	1	0
1493	chr33:5627880-5893354	0	1	0	0	0
1494	chr33:6071210-6088566	0	0	0	1	0
1495	chr33:7241101-7260538	0	1	0	1	0
1496	chr33:7587426-7605849	0	0	0	1	0
1497	chr33:7945194-7969303	0	0	0	1	0
1498	chr33:8117844-8135976	1	0	0	0	0
1499	chr33:11814760-11832450	0	0	0	1	0
1500	chr33:13596342-13610365	0	0	0	1	0
1501	chr33:17213802-17231397	0	0	0	1	0
1502	chr33:17741995-17831930	0	0	0	1	0
1503	chr33:18263437-18280710	0	0	0	1	0
1504	chr33:19745521-19769236	0	1	0	1	0
1505	chr33:22855483-22870802	0	0	0	1	0

1506	chr33:23268085-23283152	0	0	0	1	0
1507	chr33:23321720-23403572	0	0	1	0	0
1508	chr33:23421000-23521000	0	0	0	0	1
1509	chr33:23562255-23578584	0	0	0	1	0
1510	chr33:31105465-31125546	0	0	0	1	0
1511	chr33:32118630-32134770	0	0	0	1	0
1512	chr33:33464978-33480757	0	0	0	1	0
1513	chr33:33870591-33888030	0	0	0	1	0
1514	chr33:34345579-34360731	0	0	0	1	0
1515	chr34:4207926-4256266	0	1	0	1	0
1516	chr34:8262239-8279554	0	0	0	1	0
1517	chr34:12536268-12561001	0	1	0	1	0
1518	chr34:12882773-12902164	0	0	0	1	0
1519	chr34:14346798-14520557	0	1	0	0	0
1520	chr34:15145385-15175115	0	1	0	1	0
1521	chr34:15217534-15233637	0	0	0	1	0
1522	chr34:20149601-20172455	0	0	0	1	0
1523	chr34:20361397-20376550	0	0	0	1	0
1524	chr34:21566500-21666500	0	0	0	0	1
1525	chr34:23668240-23683416	0	0	0	1	0
1526	chr34:26610830-26627001	0	0	0	1	0
1527	chr34:27510736-27525882	0	0	0	1	0
1528	chr34:27749024-27769662	0	0	0	1	0
1529	chr34:28208358-28227656	0	1	0	1	0
1530	chr34:30581558-30599847	1	0	0	0	0
1531	chr34:30918847-34823181	1	1	0	1	0
1532	chr34:36913688-36931951	0	0	0	1	0
1533	chr34:40315406-40331930	0	0	0	1	0
1534	chr34:42080451-42100934	0	0	0	1	0
1535	chr34:44447229-44463597	0	0	0	1	0
1536	chr34:45086698-45114344	1	1	0	1	0
1537	chr35:3055747-3069947	0	0	0	1	0
1538	chr35:5098546-5116948	0	0	0	1	0
1539	chr35:6743927-6763362	0	0	0	1	0
1540	chr35:8148462-8168782	0	0	0	1	0
1541	chr35:10248558-10266925	0	0	0	1	0
1542	chr35:15939315-15972584	0	1	0	1	0
1543	chr35:16535000-16589000	1	0	0	0	1
1544	chr35:17102329-17119547	0	0	0	1	0
1545	chr35:18062335-18077468	0	0	0	1	0
1546	chr35:19456853-19475324	0	0	0	1	0
1547	chr35:20064567-20081884	0	0	0	1	0
1548	chr35:20306001-20325697	0	0	0	1	0
1549	chr35:27024981-27040092	0	0	0	1	0
1550	chr35:28021255-28054675	0	1	0	1	0
1551	chr35:28590087-28605460	0	0	0	1	0
1552	chr35:28863169-28880476	0	0	0	1	0
1553	chr35:28932740-28946953	0	0	0	1	0
1554	chr35:29123993-29151020	0	1	0	1	0
1555	chr36:3731712-3746936	0	0	0	1	0
1556	chr36:5742125-5757474	0	0	0	1	0
1557	chr36:7018416-7077514	0	0	1	0	0

1558	chr36:9155318-9178400	0	0	0	1	0
1559	chr36:9720373-9770373	0	0	0	0	1
1560	chr36:9988852-10012751	0	0	0	1	0
1561	chr36:13618048-13634387	0	0	0	1	0
1562	chr36:16752848-16767990	0	0	0	1	0
1563	chr36:16858375-16875844	0	0	0	1	0
1564	chr36:19512750-19529945	0	0	0	1	0
1565	chr36:20860433-20878965	0	0	0	1	0
1566	chr36:20976246-20990511	0	0	0	1	0
1567	chr36:24719443-24737764	0	0	0	1	0
1568	chr36:25960843-25979288	0	0	0	1	0
1569	chr36:27881362-27899782	0	0	0	1	0
1570	chr36:28746402-28761528	0	0	0	1	0
1571	chr36:29850403-29864656	0	0	0	1	0
1572	chr36:30605563-30638838	0	1	0	1	0
1573	chr36:30682413-30698771	0	0	0	1	0
1574	chr36:32194138-32208187	0	0	0	1	0
1575	chr36:32384758-32400038	0	0	0	1	0
1576	chr36:32533089-32557790	0	0	0	1	0
1577	chr36:33079135-33096654	0	0	0	1	0
1578	chr36:33192752-33208066	0	0	0	1	0
1579	chr37:3635260-3671648	1	0	0	0	0
1580	chr37:4501758-4520288	0	0	0	1	0
1581	chr37:4914110-4931304	0	0	0	1	0
1582	chr37:5387902-5405319	0	0	0	1	0
1583	chr37:6348122-6369309	0	1	0	1	0
1584	chr37:16080016-16098498	0	1	0	1	0
1585	chr37:17267936-17283001	0	0	0	1	0
1586	chr37:20405518-20505518	0	0	0	0	1
1587	chr37:24085594-24102882	0	0	0	1	0
1588	chr37:25505141-25530669	0	0	0	1	0
1589	chr37:26602026-26619377	0	0	0	1	0
1590	chr37:26671085-26690637	0	0	0	1	0
1591	chr37:27584574-27603180	0	0	0	1	0
1592	chr37:28375929-28390246	0	0	0	1	0
1593	chr37:29900749-29920152	0	1	0	1	0
1594	chr37:31763349-31780580	0	0	0	1	0
1595	chr37:33507508-33527175	0	0	0	1	0
1596	chr38:6165434-6179515	0	0	0	1	0
1597	chr38:6913162-7039264	0	1	0	1	0
1598	chr38:7884810-7906430	0	0	0	1	0
1599	chr38:10369367-10384564	0	0	0	1	0
1600	chr38:10437075-10506909	0	0	1	1	0
1601	chr38:11092939-11119604	0	1	0	1	0
1602	chr38:15428754-15448312	0	0	0	1	0
1603	chr38:16889950-16904151	0	1	0	1	0
1604	chr38:18603938-18619161	0	0	0	1	0
1605	chr38:20329934-20349374	0	0	0	1	0
1606	chr38:23534329-23552778	0	0	0	1	0
1607	chr38:24083547-24121103	1	0	0	1	0
1608	chr38:26203894-26263149	1	1	1	1	0
1609	chr38:26324251-26349029	0	0	0	1	0

1610	chr38:26356402-26376070	0	0	0	1	0
1611	chr38:26560200-26576890	0	1	0	1	0

* refers to CNVs identified using the 2.1 million probe aCGH experiment of Nicholas et al. 2011, but that they did not choose to report because were low confidence CNV.

Table S3. List of primers and probes used in the qPCR validation

**Gene**	***PDE4D***	***CRTC3***	***SLC6A11***
**Primer F**	TTCTGGAGGGAAGCATTTTCA	CCGTCTGGAAGACATTGTGTGT	CCTGGCAGACAGCAGGTGTA
**Primer Rev**	GTATTGATAACTGGGATGTGACATGTT	GATGACTGAGGCAAGAGAGGAAA	GGTTGGAGATAGTGCCATTGTTT
**TaqManProbe**	6FAM –CATCTGGATGAGTCGAAG-MGBNFQ	6FAM –TGCGGGCTACCTGC-MGBNFQ	6FAM –CTTCAGTAGCCAGGTGAG-MGBNFQ

Figure S1. Histogram of the number of genes in 1,000 simulated sets of regions with the same number and size of regions as the dog-specific CNV regions. In red the number of genes in the dog-specific CNV regions (*P*-value= 0.844).

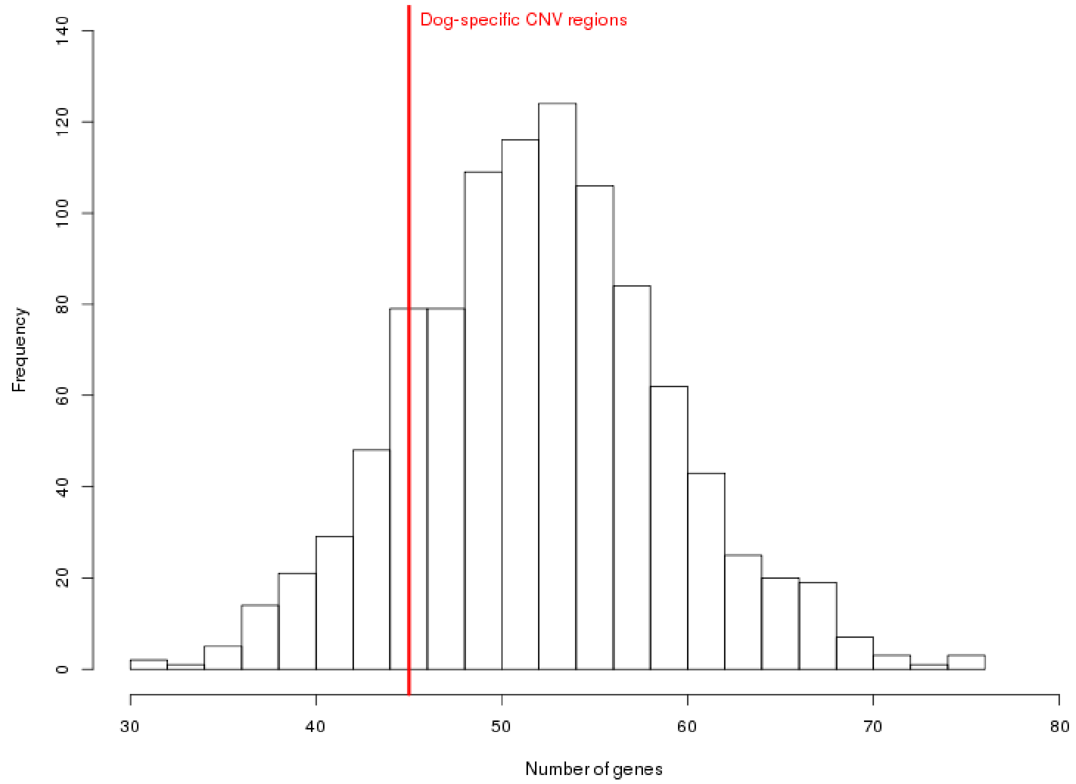

Figure S2. Histogram of the number of genes in 1,000 simulated sets of regions with the same number and size of regions as the CNV regions with only gains in wolves. In red, the number of genes in the CNV regions with only gains in wolves (P< 0.001).

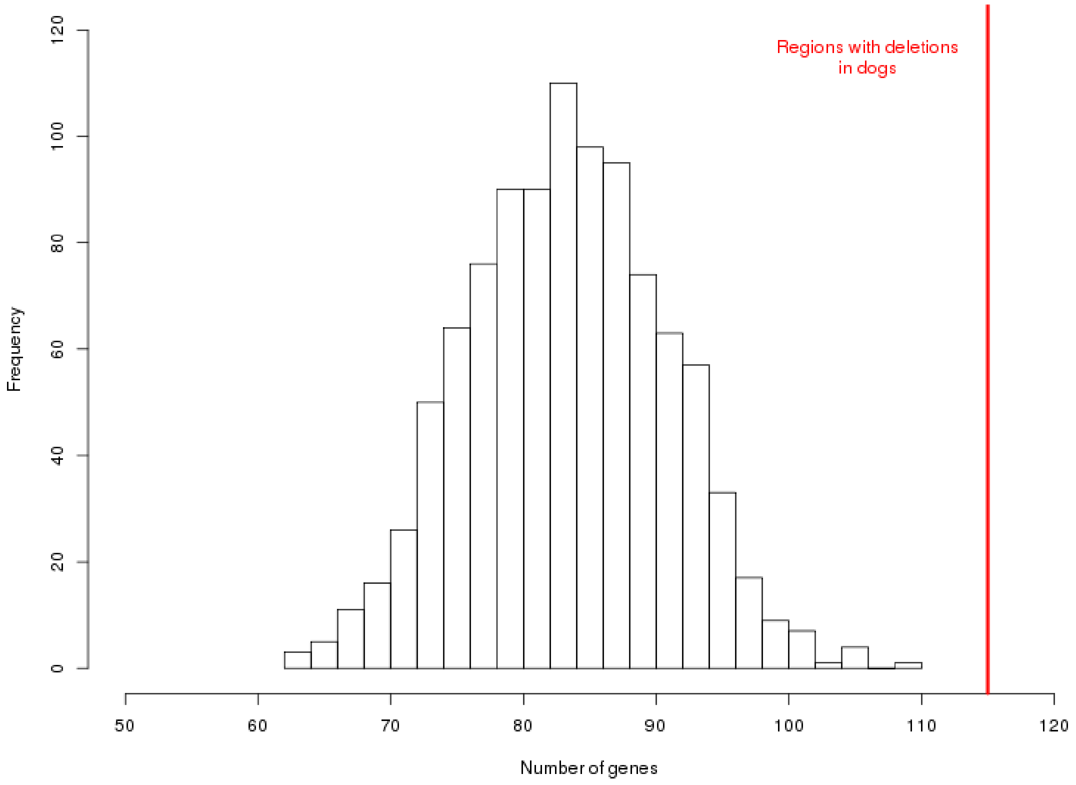

Figure S3. Enrichment analysis of GC-peaks in CNV breakpoints.
Observed to expected ratio of GC-peaks in breakpoints and in 400 bp steps away from the breakpoints.

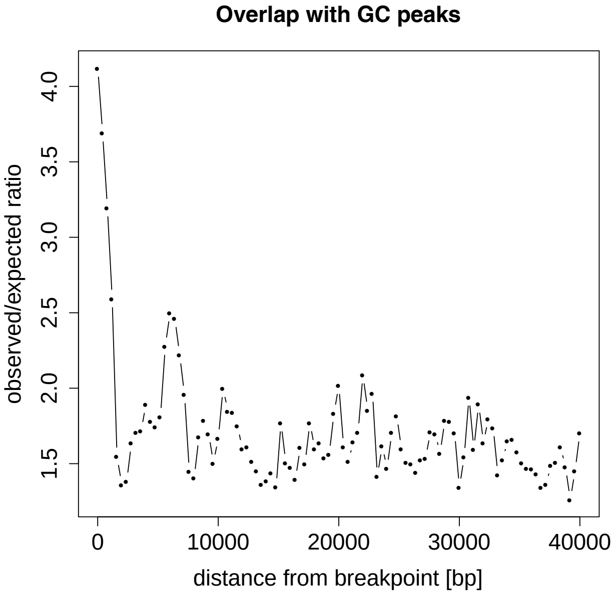

Supplement: Supplementary file 1 — Additional file 1: File containing Table S1, and S3, Figure S1, S2, S3 and their legends. (PDF 2 MB) [file 12864_2014_6138_MOESM1_ESM.pdf]
